# A large-scale metabolomics study to harness chemical diversity and explore biochemical mechanisms in ryegrass

**DOI:** 10.1038/s42003-019-0289-6

**Published:** 2019-03-04

**Authors:** Arvind K. Subbaraj, Jan Huege, Karl Fraser, Mingshu Cao, Susanne Rasmussen, Marty Faville, Scott J. Harrison, Chris S. Jones

**Affiliations:** 1 AgResearch Limited, Grasslands Research Centre, Tennent Drive, Private Bag 11008, Palmerston North, New Zealand; 2 Present Address: Institute of Agriculture and Environment, Massey University, Palmerston North, New Zealand; 3 Present Address: PepsiCo, Cork, Ireland; 4 Present Address: Feed and Forage Biosciences, International Livestock Research Institute, PO Box 5689, Addis Ababa, Ethiopia

## Abstract

Perennial ryegrass (*Lolium perenne*) is integral to temperate pastoral agriculture, which contributes most of the milk and meat production worldwide. Chemical profiles and diversity of ryegrass offer several opportunities to harness specific traits and elucidate underlying biological mechanisms for forage improvement. We conducted a large-scale metabolomics study of perennial ryegrass comprising 715 genotypes, representing 118 populations from 21 countries. Liquid/gas chromatography–mass spectrometry based targeted and non-targeted techniques were used to analyse fructan oligosaccharides, lipids, fatty acid methyl esters, polar and semi-polar compounds. Fructan diversity across all genotypes was evaluated, high- and low-sugar groups identified, and fructan accumulation mechanisms explored. Metabolites differentiating the two groups were characterised, modules and pathways they represent deduced, and finally, visualisation and interpretation provided in a biological context. We also demonstrate a workflow for large-scale metabolomics studies from raw data through to statistical and pathway analysis. Raw files and metadata are available at the MetaboLights database.

## Abstract (web summary)

Arvind Subbaraj et al. present the ryegrass metabolome, derived from 715 genotypes representing 118 populations of *Lolium perenne* from 21 countries. They analyze fructan diversity, identify high- and low-sugar groups, and explore biochemical modules and pathways that discriminate the phenotypes.

## Introduction

Perennial ryegrass (*Lolium perenne* L. Family: *Poaceae*)^1^ supports most of the milk and meat production worldwide^2^. Geno-phenotypic characteristics of ryegrass are therefore critical in determining feed quality for the animal^3^, degradation in the rumen^4^ and livestock production responses^5–7^. Consequently, breeding techniques^8–11^ are employed to produce cultivars with desirable traits such as high water soluble carbohydrate content, neutral detergent fibre, crude protein content and digestibility, in addition to forage yield, seed yield, pest and disease resistance^12,13^. Of special interest and relevance to this study are the high-sugar cultivars, which have elevated levels of fructans. Fructans are the major storage carbohydrate in ryegrass, and are made up of varying degrees and complexities of linear or branched fructose polymers^14^, denoted by the degree of polymerisation (DP). DP directs fructan accumulation and thereby the total sugar content of these high-sugar cultivars^15^. High-sugar cultivars are proposed to increase milk and meat production through enhanced protein utilisation by ruminants^16^. In addition, lipid composition of ryegrass affects quality of animal products^12^, and secondary metabolites possess anti-parasitic activity in ruminants^17^. We hypothesised that chemical diversity of ryegrass, especially fructan content, offers opportunities to harness variation in these traits into cultivars for improved ruminant performance and novel product characteristics and, exploring underlying biochemical mechanisms of high-sugar grasses, in addition to a better mechanistic understanding of fructan accumulation, will also help decipher major changes in primary and secondary metabolism.

Metabolomics^18,19^ provides a snapshot of chemical diversity, enabling metabotypic classification of genotypes^20–22^ and in conjunction with other –omics sciences^23^, a better understanding of biological mechanisms^18^. The advent of advanced bio/cheminformatic tools and techniques^24^ have since propelled metabolomics towards system-level evaluations via data-fusion^25^ and pathway mapping^26^. We conducted a mass spectrometry based metabolomics study of 5 clonal replicates of 715 ryegrass genotypes (3575 plants), representing 118 populations from 21 countries (Supplementary Figure 1). Fructans, fatty acid methyl esters (FAMEs), lipids, polar and semi-polar compounds were analysed using ultra-high-performance liquid chromatography (U)HPLC and gas chromatography–mass spectrometry (GC–MS) systems. The objectives of the current study were to verify and demonstrate quality control measures undertaken for big metabolomics data, evaluate diversity of ryegrass genotypes for fructan/sugar content, and thereby identify high- and low-sugar plant genotypes under New Zealand climatic conditions, determine the role of DP of fructans in directing total sugar content, and finally elucidate potential metabolic variation between high- and low-sugar grasses in the context of data from other analytical streams (lipids, FAMEs, polar and semi-polar compounds).

Constant monitoring and post-run evaluation of quality control parameters accounted for technical variation in samples, and where these parameters were not met, batches were re-run following instrument calibration. The quality control procedures adopted here were therefore appropriate for large-scale metabolomics studies, rendering reliable data for downstream processing. The sum of low- (3–5), mid- (10–12) and high- (18–20) DP fructans was used as a measure of total sugar content, and of the 715 genotypes surveyed, 39 high- and 31 low-sugar genotypes were identified. High-DP fructans contributed significantly more to the total sugar content, measured as hexose units, in high-sugar grasses. A negative correlation between high- and low-DP fructans in the high-sugar group, further identified 11 genotypes which had greater high-DP content than a reference high-sugar genotype (Aberdart). These results, in addition to immediate inclusion in breeding exercises, offer a better understanding of fructan accumulation in high-sugar grasses, and subsequently ample scope for genetic improvement. Between high- and low-sugar grasses, major differences in primary metabolism were observed, with most lipid classes and fatty acids significantly higher in the low-sugar group. Differences in secondary metabolism were also noticed, where high-sugar grasses recorded lower concentrations of flavonoids and lignins. Identification of compounds and mapping them to metabolic pathways, successfully led to visualisation of a biochemical snapshot of high-sugar grasses.

## Results

### Quality control monitoring and evaluation

In large-scale metabolomics studies, demonstration of quality control monitoring and verification of quality control parameters is a prerequisite which accounts for technical variation, and affects data quality and thereby subsequent interpretation. Drifts in mass accuracy and retention time of the internal standard in quality control samples indicates technical variation between batches of samples. Here, drifts in mass accuracy and retention times of the internal standard 2′,7′-Dichlorofluorescein in quality control samples of the semi-polar stream (positive ionisation mode), across all 36 batches was demonstrated (Supplementary Figure 2). Mass accuracy was within the ±5 ppm threshold (Supplementary Figure 2A), and retention time drifts were within ±0.2 min from the median (Supplementary Figure 2B). Quality control monitoring for the lipid and polar streams also generated identical results.

A post-run evaluation of run-order effects was also conducted immediately after each batch was completed. Supplementary Figure 3 shows an exemplar principal component analysis (PCA) of a single batch of samples classified based on run-order, where Supplementary Figure 3A shows no significant run-order effect, while Supplementary Figure 3B shows a notable run-order effect. In this case, the batch representing Supplementary Figure 3A was proceeded to the super batch, whereas that representing Supplementary Figure 3B was re-run. Since quality control samples were not representative of the sample set, they clustered separately from the samples.

### Chemical diversity

*Fructan content*: A typical total ion chromatogram of a sample run for fructan measurement, depicting low (3–5), mid (10–12) and high (18–20) DP ranges, is shown in Supplementary Figure 4. These ranges were used to measure the total sugar content of the sample. Samples in a single batch for fructan/sugar estimates before (Supplementary Figure 5A) and after (Supplementary Figure 5B) normalisation by a linear trend are shown in Supplementary Figure 5. Likewise, estimates between all 36 batches of samples, before (Supplementary Figure 6A) and after (Supplementary Figure 6B) normalisation for batch-effects, are shown in Supplementary Figure 6. The resultant data matrix, obtained after these normalisation procedures was used for classification of genotypes based on total sugar content.

**Fig. 1:**
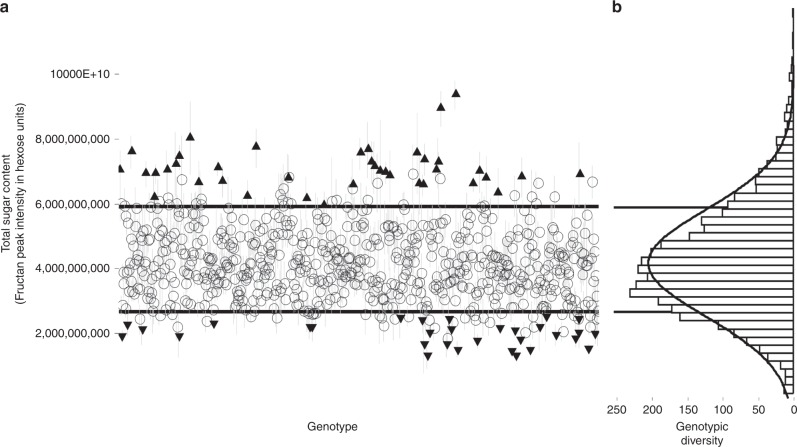
Diversity of ryegrass genotypes (open circle, black up pointing triangle, black down pointing triangle) (**a**) based on total sugar content ±SE (*n* = 5), with black up pointing triangle denoting the top 10% (high-sugar group) and, black down pointing triangle denoting the bottom 10% (low-sugar group) that fulfilled the two-tier criterion, and **b** showing the genotypic diversity based on total sugar content, relative to a normal distribution curve

Normal distribution of ryegrass genotypic diversity based on total sugar content is shown in Fig. 1. Of the top 10%, only genotypes that fulfilled our two-tier criterion of having a minimum of three replicates in the top 10% of the whole sample set (3575), were classified as the high-sugar group (*n* = 133 samples; *p* = 0.0001; Tukey’s HSD). Based on these conditions, the high-sugar group comprised 39 genotypes, of which 19 had genetic lineage to New Zealand (Supplementary Table 1). As anticipated, genotypes of the high-sugar grasses Aberdart and Aurora, bred in the UK^27^, were present in this group. The remainder of this pool was made up of genotypes from Netherlands, Denmark, Australia, Slovakia, Tunisia and Germany (Supplementary Table 1). Likewise, the bottom 10%, low-sugar group (*n* = 106), comprised 31 genotypes, with 14 being of New Zealand origin and the remainder of genotypes from Tunisia (Supplementary Table 1).

**Fig. 2:**
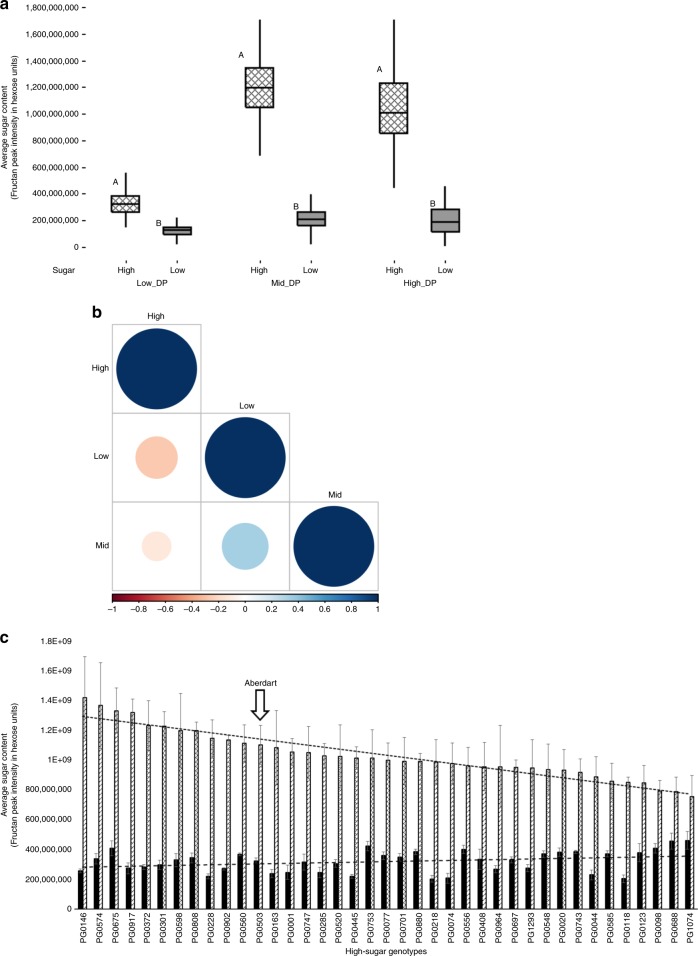
**a** Boxplots of total sugar content between high- and low-sugar groups, distributed across average peak intensities of low (DP3–5), mid (DP10–12) and high (DP18–20) degree of polymerisation (DP) of fructans. Patterned and shaded boxes denote high- and low-sugar groups, respectively. Different upper case letter codes presented between the two groups, indicate significantly different (*p* < 0.05) values by Tukey’s HSD. **b** Correlation analysis (Pearson method) between low-, mid- and high-DP fructans within the 39 high-sugar genotypes. Positive correlations are displayed in blue and negative correlations in red colour. Colour intensity and the size of the circle are proportional to the correlation coefficients. Legend colour at the bottom shows the correlation coefficients and the corresponding colours. **c** Comparison of average peak intensities ± SE of high- (Patterned bars) and low-DP (shaded bars) fructans across the 39 high-sugar genotypes, along with respective linear regression trends. The high-sugar genotype Aberdart is marked as the current standard. Data underlying the plots in **c** are available in Supplementary Data 3

*Fructan accumulation in high- vs. low-sugar grasses*: Fructan accumulation was significantly higher (*p* = 0.0001; Tukey’s HSD) in high-sugar grasses across all DPs (Fig. 2a). However, within the high-sugar group, the contribution of high- and mid-DPs to the total sugar content was more prominent than low-DP (Fig. 2a). A negative correlation between high- and low-DP was evident within high-sugar genotypes (Fig. 2b), which when extended to the 39 genotypes, revealed the ones with higher high- to low-DP ratios (Fig. 2c). Provided genotypes with high-DP levels are preferred within the high-sugar group, 11 genotypes with greater high-DP content than the current standard for high-sugar genotypes (Aberdart) were identified (Fig. 2c).

**Fig. 3:**
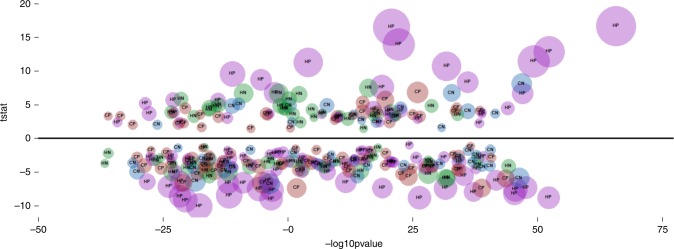
Cloudplot of *t* stat and −log10 *p* values of metabolic features from the HILIC and C18 streams (positive- and negative-ionisation modes), significantly different between high- and low-sugar groups, based on *t* tests with a false-discovery rate cut-off of *p* < 0.05. A positive *t* stat value indicates high- > low-sugar group, whereas a negative value indicates high- < low-sugar group. Cloud size represents the magnitude of –log10 *p* value. HP (Purple), HN (Green), CP (Brown) and CN (Blue) represent different analytical streams corresponding to HILIC positive, negative and C18 positive and negative, respectively

*Polar and semi-polar compounds*: Following data processing, the final data matrices from the HILIC streams had 222 (positive) and 198 (negative), and those from the C18 streams had 175 (positive) and 152 (negative) metabolic features, respectively. Data for high- and low-sugar groups were compared, and of the total 747 features, 293 were significantly different between the two groups, based on *t* tests with a false-discovery rate cut-off of *p* < 0.05 (Fig. 3; Supplementary Data 1). Multivariate analysis with PCA for each analytical stream, failed to discriminate the two groups (Supplementary Figure 7).

An overview of the discriminating features (Fig. 3) showed that polar compounds from HILIC positive and negative-ionisation streams, indicative of primary metabolism, demonstrated maximum variation between the high- and low-sugar groups, compared to semi-polar compounds from C18 positive- and negative-ionisation streams, largely representative of secondary metabolism. The HILIC positive stream accounted for 104 significantly different features, 35 of which were higher in the high-sugar group (Fig. 3). The HILIC negative stream revealed 64 significantly different features, of which 29 were higher in the high-sugar group (Fig. 3). C18 positive and negative streams had 80 and 45 discriminating features, of which 29 and 16 respectively, were significantly higher in the high-sugar group (Fig. 3).

**Fig. 4:**
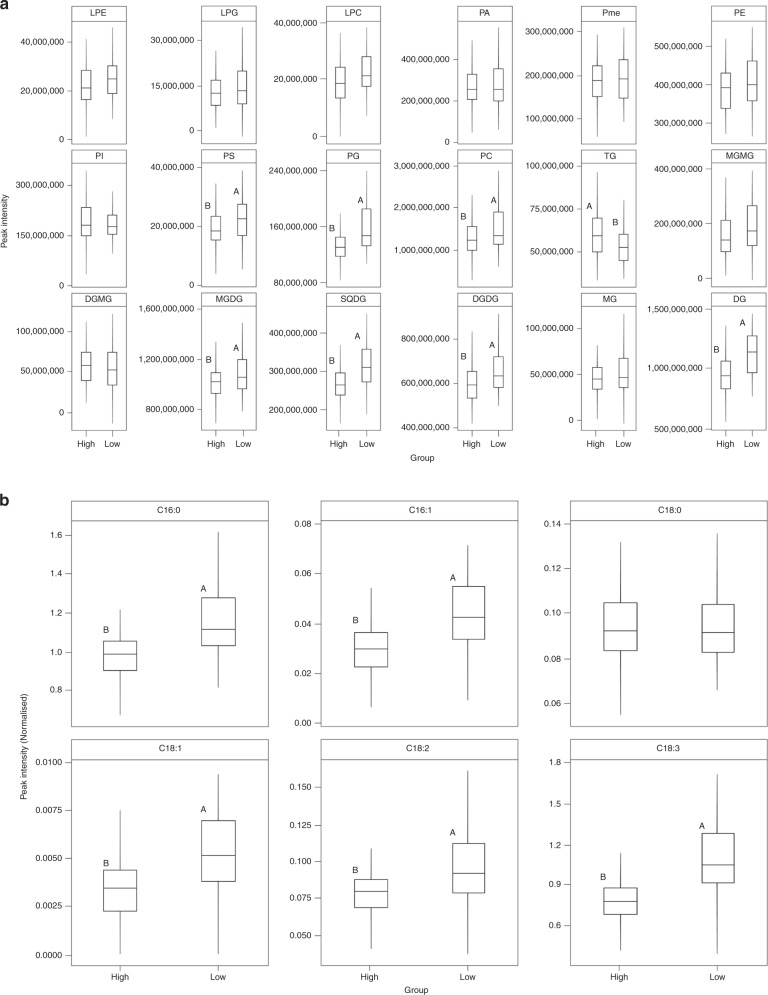
Boxplots of peak intensities of lipid classes identified by **a** the non-targeted lipidomics method and **b** normalised peak intensities of fatty acid methyl esters (FAMEs) identified by the targeted method, between the high- and low-sugar groups. Different upper case letter codes, where presented between the two groups, indicate significantly different (*p* < 0.05) values by Tukey’s HSD. LPE lysophosphatidylethanolamine, LPG lysophosphatidylglycerol, LPC lysophosphatidylcholine, PA phosphatidic acid, Pme phosphatidylmethanol, PE phosphatidylethanolamine, PI phosphatidylinositol, PS phosphatidylserine, PG phosphatidylglycerol, PC phosphatidylcholine, TG triglyceride, MGMG monogalactosylmonoacylglycerol, DGMG digalactosylmonoacylglycerol, MGDG monogalactosyldiacylglycerol, SQDG sulfoquinovosyldiacylglycerol, DGDG digalactosyldiacylglycerol, MG monoglyceride, DG diglyceride. C16:0, C16:1, C18:0, C18:1, C18:2 and C18:3 refer to fatty acids with their respective number of carbon atoms and double bonds

*Lipids*: Major lipid classes identified by the non-targeted lipidomics method (Fig. 4a) and the targeted FAMEs method (Fig. 4b) are shown in Fig. 4. Taken together, concentrations of phosphatidylserine (*p* = 0.005), phosphatidylglycerol (*p* = 0.0001), phosphatidylcholine (*p* = 0.001), monogalactosyldiacylglycerol (*p* = 0.004), sulfoquinovosyldiacylglycerol (*p* = 0.0001), digalactosyldiacylglycerol (*p* = 0.009), diglycerides (*p* = 0.0001) and fatty acids C16:0 (*p* = 0.0001), C16:1 (*p* = 0.0001), C18:1 (*p* = 0.0001), C18:2 (*p* = 0.0001) and C18:3 (*p* = 0.0001) were higher in the low-sugar group (Fig. 4; Tukey’s HSD). Lysophosphatidylethanolamine, lysophosphatidylglycerol, lysophosphatidylcholine, phosphatidic acid, phosphatidylmethanol, phosphatidylethanolamine, phosphatidylinositol, monogalactosylmonoacylglycerol, digalactosylmonoacylglycerol, monoglycerides and fatty acid C18:0, were not significantly different between the two groups (*p* > 0.05). Triglycerides alone were in higher concentrations in the high-sugar group (*p* = 0.002; Tukey’s HSD; Fig. 4). Overall, 100 lipid species belonging to 18 lipid classes were identified by the non-targeted lipidomics stream (Supplementary data 2).

*Compound identification*: Compounds identified in the current study based on matching with a local library of authentic standards, de novo matching with public domain mass spectral databases, and/or the *Mummichog* programme, are presented in Table 1. A matching with the local library was given maximum confidence (Level 1), followed by matching with spectral databases (Level 2). Supplementary Figure 8 shows one such match of quinic acid/quinate (C_7_H_12_O_6_; KEGG ID—C00296), where the extracted ion chromatogram for the parent mass [M–H]^−^ *m/z* 191.0554 from a sample in HILIC-negative ionisation stream co-elutes with those for diagnostic fragments *m/z* 173.0449 (C_7_H_9_O_5_), 127.0392 (C_6_H_7_O_3_), 111.0443 (C_5_H_3_O_3_) and 93.0336 (C_6_H_5_O) (Supplementary Figure 8A). Also, mass spectra of diagnostic fragments *m/z* 93.0336, 111.0078, and 127.0392 (Supplementary Figure 8B) matched with corresponding spectra in the public domain MS database METLIN^28^ (Supplementary Figure 8C).

**Table 1:** Summary of compounds identified by matching with a local library of authentic standards, public domain mass spectral databases and/or *Mummichog*, with their respective KEGG IDs, analytical stream, univariate statistics and level of confidence in identification

KEGG ID	Name		Stream	Univariate statistics			Identification				
				Fold	*t* stat	AUC	Confidence	Library	Database	*Mummichog*	
								Parent [M ± H]^±^	Diagnostic *m/z*	*m/z*	Tentative match
C00317	Amylopectin	↑	HP	4.62	17.11	1.0	Level 3			867.2384	M + K[1+]
C00208	Maltose	↑	HP	2.49	10.15	0.9	Level 3			307.1023, 325.1129, 381.0794	M–H_4_O_2_ + H[1+], M–H_2_O + H[1+], M + K[1+]
C01083	alpha,alpha-Trehalose	↑	HP	2.49	10.15	0.9	Level 3			307.1023, 325.1129, 381.0794	M–H_4_O_2_ + H[1+], M–H_2_O + H[1+], M + K[1+]
C01235	Galactinol	↑	HP	2.49	10.15	0.9	Level 3			307.1023, 325.1129, 381.0794	M–H_4_O_2_ + H[1+], M–H_2_O + H[1+], M + K[1+]
C04332	6,7-Dimethyl-8-(1'-D-Ribityl)Lumazine	↑	HP	2.49	10.15	0.9	Level 3			325.1129	M[1+]
C00117	d-Ribose 5'-Phosphate	↑	HP	2.04	9.60	0.8	Level 3			145.0496	M–HCOOK + H[1+]
C00124	d-Galactose	↑	HP	2.04	9.60	0.8	Level 3			145.0496	M–H_4_O_2_ + H[1+]
C00137	Myo-inositol	↑	HN	2.04	9.60	0.8	Level 1	179.0556			
C00221	Beta-d-Glucose	↑	HP	2.04	9.60	0.8	Level 3			145.0496	M–H_4_O_2_ + H[1+]
C00231	d-Xylulose 5'-Phosphate	↑	HP	2.04	9.60	0.8	Level 3			145.0496	M–HCOOK + H[1+]
C00267	Alpha-d-Glucose	↑	HP	2.04	9.60	0.8	Level 3			145.0496	M–H_4_O_2_ + H[1+]
C00962	Beta-d-Galactose	↑	HP	2.04	9.60	0.8	Level 3			145.0496	M–H_4_O_2_ + H[1+]
C00966	2-Dehydropantoate	↑	HP	2.04	9.60	0.8	Level 3			145.0496	M[1+]
C01077	O-acetyl-l-homoserine	↑	HP	2.04	9.60	0.8	Level 2		102.0550, 74.0609	90.0556, 116.071, 118.0867, 145.0496, 180.0867	M–C_3_H_4_O_2_ + H[1+], M–HCOOH + H[1+], M–CO_2_ + H[1+], M-NH_3_ + H[1+], M + H_2_O + H[1+]
C01112	d-Arabinose 5-Phosphate	↑	HP	2.04	9.60	0.8	Level 3			145.0496	M–HCOOK + H[1+]
C01825	l-Galactose	↑	HP	2.04	9.60	0.8	Level 3			145.0496	M–H_4_O_2_ + H[1+]
C01906	Hamamelose	↑	HP	2.04	9.60	0.8	Level 2		181.0714, 163.0608	145.0496	M–H_4_O_2_ + H[1+]
C02336	Beta-d-Fructose	↑	HP	2.04	9.60	0.8	Level 3			145.0496	M–H_4_O_2_ + H[1+]
C03906	Beta-l-Arabinose 1-Phosphate	↑	HP	2.04	9.60	0.8	Level 3			145.0496	M–HCOOK + H[1+]
C04236	(2S)-2-Isopropyl-3-Oxosuccinate	↑	HP	2.04	9.60	0.8	Level 3			101.0237, 127.0394, 145.0496	M–C_3_H_4_O_2_ + H[1+], M–HCOOH + H[1+], M–CO + H[1+]
C06006	(S)-2-Aceto-2-Hydroxybutanoate	↑	HP	2.04	9.60	0.8	Level 3			145.0496	M[1+]
C00555	4-Aminobutyraldehyde	↑	HP	1.70	6.95	0.8	Level 3			127.0394	M + K[1+]
C01210	N-Methylethanolamine Phosphate	↑	HP	1.70	6.95	0.8	Level 3			109.0288, 127.0394	M–HCOOH + H[1+], M–CO + H[1+]
C02351	1,2-Benzoquinone	↑	HP	1.70	6.95	0.8	Level 2		109.0282, 81.0342	109.0288, 81.034, 127.0394	M + H[1+], M-CO + H[1+], M + H_2_O + H[1+]
C00296	Quinate/Quinic acid	↑	HN	2.26	6.72	0.8	Level 2		191.0554		
C00111	Glycerone Phosphate	↑	HP	1.66	6.65	0.8	Level 3			125.0003, 85.029	M–CO_2_ + H[1+], M–HCOOK + H[1+]
C00661	d-Glyceraldehyde 3-Phosphate	↑	HP	1.66	6.65	0.8	Level 3			125.0003, 85.029	M–CO_2_ + H[1+], M–HCOOK + H[1+]
C01234	1-Aminocyclopropane-1-Carboxylate	↑	HP	1.66	6.65	0.8	Level 3			102.0554, 74.0606, 84.0449, 85.029, 120.066	M + H[1+], M–CO + H[1+], M–H_2_O + H[1+], M–NH_3_ + H[1+], M + H_2_O + H[1+]
C02631	2-Isopropylmaleate	↑	HP	1.66	6.65	0.8	Level 3			85.029	M–C_3_H_4_O_2_ + H[1+]
C13482	Phosphodimethylethanolamine	↑	HP	1.66	6.65	0.8	Level 3			97.029, 85.029	M–C_3_H_4_O_2_ + H[1+], M + 2 H[2+]
C17759	1-O-Feruloyl-Beta-d-Glucose	↑	HN	1.53	4.45	0.7	Level 3			337.0929, 371.0984	M–H_2_O-H[−], M–H + O[−]
C10883	(+)-Sesamolin	↑	HN	1.72	3.43	0.7	Level 3			371.0984, 390.0726	M(^37^Cl)–H[−], M + Na-2H[−]
C00152	l-Asparagine	↓	HP	1.77	−2.54	0.6	Level 1	133.0614			
C09315	Umbelliferone	↓	CN	2.15	−2.75	0.6	Level 2		161.0237, 162.0272	161.0239, 202.0505	M–H[−], M + ACN-H[−]
C01460	Vitexin	↓	CN	1.65	−2.75	0.6	Level 2		431.0976, 311.0555	447.0929, 477.1036	M–H + O[−], M + HCOO[−]
C01714	Isovitexin	↓	CN	1.65	−2.75	0.6	Level 3			447.0929, 477.1036	M-H + O[−], M + HCOO[−]
C01821	Isoorientin	↓	CN	1.65	−2.75	0.6	Level 3			447.0929, 506.1017	M(^13^C)–H[−], M + CH_3_COO[−]
C08604	Chrysanthemin	↓	CN	1.65	−2.75	0.7	Level 3			447.0929, 463.088, 493.1007, 506.1017	M–H[−], M–H + O[−], M + HCOO[−], M + CH_3_COO[−]
C10114	Orientin	↓	CN	1.65	−2.75	0.6	Level 2		447.0925, 327.0499	447.0929, 506.1017	M(^13^C)–H[−], M + CH_3_COO[−]
C12137	Pelargonidin-3-O-Beta-D-Glucoside	↓	CN	1.65	−2.75	0.6	Level 3			447.0929, 477.1036	M–H + O[−], M + HCOO[−]
C16298	Cyanidin 5-O-Beta-D-Glucoside	↓	CN	1.65	−2.75	0.6	Level 3			447.0929, 506.1017	M(^13^C)–H[−], M + CH_3_COO[−]
C01617	Taxifolin	↓	CP	1.77	−2.85	0.6	Level 3			287.0548	M–H_2_O + H[1+]
C05631	Eriodictyol	↓	CP	1.77	−2.85	0.6	Level 3			287.0548	M[1 + ]
C05909	Leucodelphinidin	↓	CP	1.77	−2.85	0.6	Level 3			287.0548	M–H_4_O_2_ + H[1+]
C00327	l-Citrulline	↓	HP	1.66	−4.25	0.7	Level 2		176.1038, 159.0772	159.0766	M–NH_3_ + H[1+]
C00065	l-Serine	↓	HP	1.51	−4.41	0.6	Level 1	106.0506			
C01092	8-Amino-7-Oxononanoate	↓	CN	1.75	−4.91	0.7	Level 3			223.0608	M + K-2H[−]
C02666	Coniferyl Aldehyde	↓	CN	1.75	−4.91	0.7	Level 2		177.0550, 162.0321	193.0501, 223.0608	M–H + O[−], M + HCOO[−]
C05610	Sinapoyl Aldehyde	↓	CN	1.75	−4.91	0.7	Level 3			207.0658, 223.0608	M–H[−], M–H + O[−]
C03319	DTDP-Beta-l-Rhamnose	↓	HP	2.15	−5.04	0.8	Level 3			501.0645	M–HCOOH + H[1+]
C11907	DTDP-4-Dehydro-6-Deoxy-Alpha-D-Glucopyranose	↓	HP	2.15	−5.04	0.8	Level 3			501.0645	M–CO_2_ + H[1+]
C00021	S-Adenosyl-l-Homocysteine	↓	CN	1.75	−5.71	0.7	Level 3			385.1138	M(^34^S)–H[−]
C01175	1-O-Sinapoyl-Beta-d-Glucose	↓	CN	1.75	−5.71	0.7	Level 2		385.1133, 205.0499, 191.0554	385.1138, 367.103	M–H[−], M–H_2_O–H[−]
C16827	1-O-(4-Coumaroyl)-Beta-d-Glucose	↓	CN	1.75	−5.71	0.7	Level 3			341.0875, 371.098, 385.1138	M–H + O[−], M + HCOO[−], M + CH_3_COO[−]

HP, HN, CP and CN denote different analytical streams corresponding to HILIC positive, negative and C18 positive and negative, respectively; a positive *t* stat value indicates high- > low-sugar group (↑), whereas a negative value indicates high- < low-sugar group (↓); area under the curve (AUC) is a summary statistic for receiver–operator characteristic (ROC) curves, and denotes the trade-off between the specificity and sensitivity of a compound to enable binary classification of the two groups. On a rough scale, AUC values of 0.9–1.0 = excellent, 0.8–0.9 = good, 0.7–0.8 = fair, 0.6–0.7 = poor and 0.5–0.6 = fail, denote respective powers of the compound to direct binary classification^79^; compounds with matches in the library, spectral database or *Mummichog* were scored with 1, 2 or 3 levels of confidence, respectively^44^.

Metabolic features (*m/z*) and their tentative matches used by *Mummichog* for identification are presented in Table 1. As is evident, a single ion mass may relate to many compounds or a group of masses may relate to one compound. Nevertheless, the compound classes identified by *Mummichog* provides sufficient information to interrogate the data further towards a higher level of confidence (Level 2). In the case of coniferyl aldehyde (Table 1), which was initially identified by *Mummichog* using *m/z* 193.0501 and 223.0608 corresponding to M–H + O[−] and M + HCOO[−], respectively, the parent mass of coniferyl aldehyde (C_10_H_10_O_3_), [M–H]^−^ *m/z* 177.0550, and its diagnostic fragment *m/z* 162.0321 (C_9_H_6_O_3_) were subsequently queried in the sample and spectral databases. As explained in Supplementary Figure 8, co-elution of the extracted ion chromatograms of these features, and matching of these spectra in the sample with corresponding spectra in MassBank^29^, with a mass error of <5 ppm, led to tentative identification of coniferyl aldehyde with Level 2 confidence. Even so, redundancies in identifications by *Mummichog*, for example, d-Ribose 5-Phosphate, alpha-d-Galactose, beta-d-Glucose, beta-d-Galactose etc., all identified for *m/z* 145.0496, only conform to the identification of monosaccharides or monosaccharide phosphates in the high-sugar group. Therefore, *Mummichog* results helped identify potential leads for compound identification, albeit with a lower level of confidence.

**Fig. 5:**
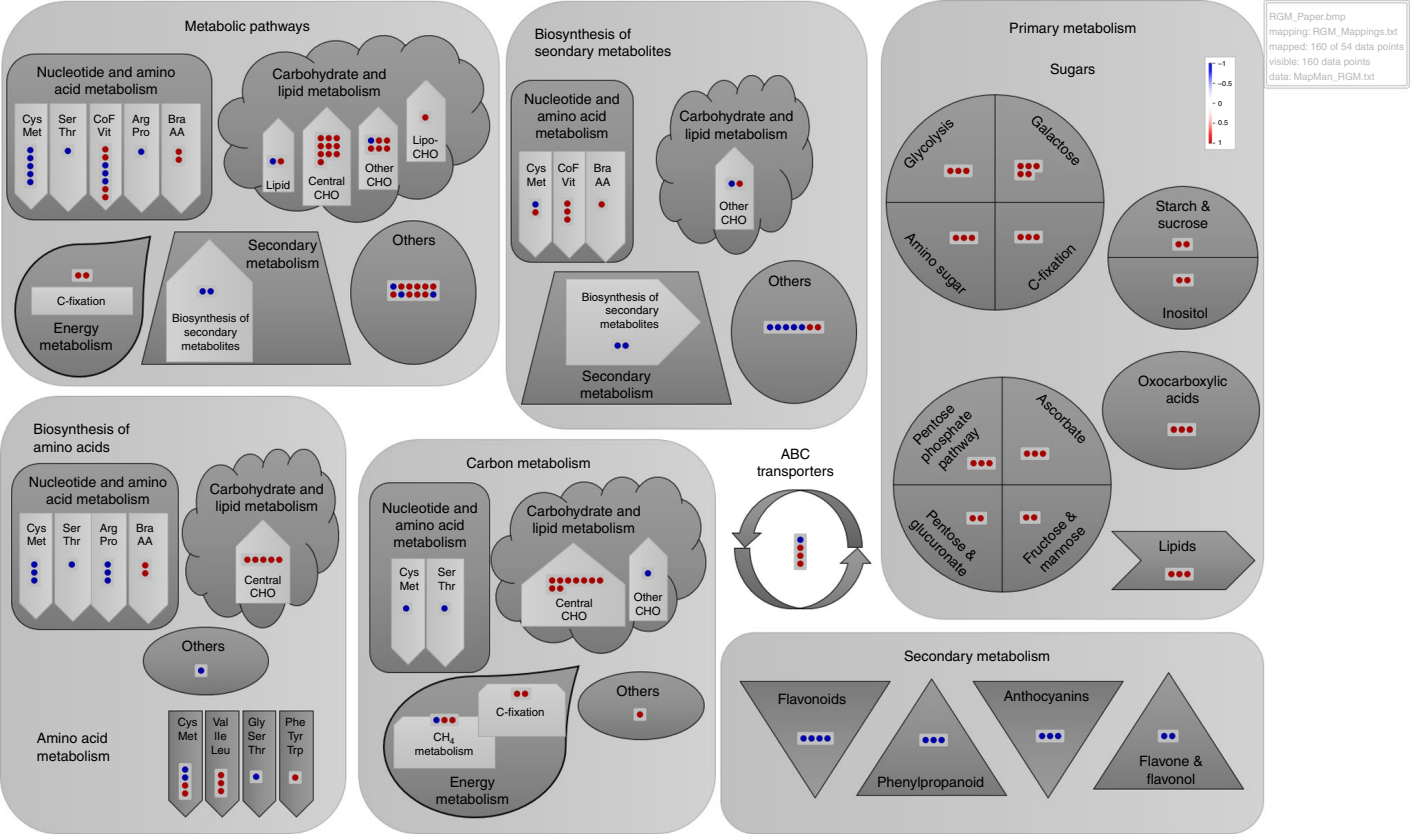
Pictorial representation of biochemical activity of high-sugar grasses in MapMan^77^ (Ver 3.6.0RC1; copyright of the Max–Planck-Institute for Molecular Plant Physiology, Golm, Germany), depicting KEGG pathways and modules involving the compounds identified in Table 1, with rice pathways (osa) as a reference (Table 2). Dots represent compounds identified in respective modules, where red dots denote compounds with positive *t* stat value (high- > low-sugar group), and blue dots denote compounds with negative *t* stat value (high- < low-sugar group). CHO: carbohydrates, Bra-AA: branched chain amino acids, CoF and Vit: cofactor and vitamins, ABC: ATP-binding cassette

*Modules and pathway analysis*: Of all identified compounds input into KEGG Mapper (Table 1) with rice pathways (osa) as a reference, 29 mapped to metabolic pathways (osa01100), 23 to the biosynthesis of secondary metabolites (osa01110), 8 to the biosynthesis of amino acids (osa01230), 6 to carbon metabolism (osa01200) and the rest to miscellaneous pathways related to primary and secondary metabolism (Table 2). Each pathway is characterised by several modules, and each module comprises several compounds and corresponding reactions. Redundancies in a single compound represented in multiple modules, and a single module accommodating several identified compounds, was observed (Table 2). A pictorial representation of compounds mapped to respective pathways/modules in the context of high-sugar grasses is depicted in Fig. 5.

**Table 2:** KEGG pathway modules and hierarchical bin structure for MapMan style representation of compounds identified in Table 1, matched with rice reference pathways (osa) using KEGG Mapper

Bin code	Name	Module	Reaction	Compound	KEGG ID	
1	Metabolic pathways					
1.1	*Nucleotide and amino acid metabolism*					
1.1.1	*Cysteine and methionine metabolism*					
1.1.1.1	Methionine degradation	M00035	S-Adenosyl-l-Methionine → l-Homocysteine	S-Adenosyl-l-Homocysteine	C00021	↓
1.1.1.2	Methionine degradation	M00035	l-Serine → l-Cystathionine	l-Serine	C00065	↓
1.1.1.3	Cysteine biosynthesis	M00609	Methionine → Cysteine	S-Adenosyl-l-Homocysteine	C00021	↓
1.1.1.4	Cysteine biosynthesis	M00021	Serine → Cysteine	l-Serine	C00065	↓
1.1.1.5	Cysteine biosynthesis	M00338	Homocysteine + Serine → Cysteine	l-Serine	C00065	↓
1.1.2	*Serine and threonine metabolism*					
1.1.2.1	Serine biosynthesis	M00020	Glycerate-3P → Serine	l-Serine	C00065	↓
1.1.3	*Cofactor and vitamin biosynthesis*					
1.1.3.1	Ascorbate degradation	M00550	Ascorbate → d-Xylulose 5-Phosphate	d-Xylulose 5-Phosphate	C00231	↑
1.1.3.2	Pantothenate biosynthesis	M00119	Valine/l-Aspartate → Pantothenate	2-Dehydropantoate	C00966	↑
1.1.3.3	Biotin biosynthesis	M00123	Pimeloyl-ACP/CoA → Biotin	8-Amino-7-Oxononanoate	C01092	↓
1.1.3.4	Biotin biosynthesis, BioI pathway	M00573	Long-chain-acyl-ACP → Pimeloyl-ACP → Biotin	8-Amino-7-Oxononanoate	C01092	↓
1.1.3.5	Biotin biosynthesis, BioW pathway	M00577	Pimelate → Pimeloyl-CoA → Biotin	8-Amino-7-Oxononanoate	C01092	↓
1.1.3.6	Ascorbate biosynthesis	M00114	Glucose 6-Phosphate → Ascorbate	l-Galactose	C01825	↑
1.1.3.7	Riboflavin biosynthesis	M00125	GTP → Riboflavin/FMN/FAD	6,7-Dimethyl-8-(d-Ribityl)Lumazine	C04332	↑
1.1.4	*Arginine and proline metabolism*					
1.1.4.1	Urea cycle	M00029		l-Citrulline	C00327	↓
1.1.5	*Branched-chain amino acid metabolism*					
1.1.5.1	Valine/Isoleucine biosynthesis	M00019	Pyruvate → Valine/2-Oxobutanoate → Isoleucine	(S)-2-Aceto-2-Hydroxybutanoate	C06006	↑
1.1.5.2	Isoleucine biosynthesis	M00570	Threonine → 2-Oxobutanoate → Isoleucine	(S)-2-Aceto-2-Hydroxybutanoate	C06006	↑
1.2	*Carbohydrate and lipid metabolism*					
1.2.1	*Lipid metabolism*					
1.2.1.1	Ceramide biosynthesis	M00094		l-Serine	C00065	↓
1.2.1.2	Inositol phosphate metabolism	M00131	Ins(1,3,4,5)P4 → Ins(1,3,4)P3 → Myo-inositol	Myo-Inositol	C00137	↑
1.2.2	*Other carbohydrate metabolism*					
1.2.2.1	Photorespiration	M00532		l-Serine	C00065	↓
1.2.2.2	Nucleotide sugar biosynthesis	M00554	Galactose → UDP-Galactose	d-Galactose	C00124	↑
1.2.2.3	Galactose degradation, Leloir pathway	M00632	Galactose → Alpha-D-Glucose 1-Phosphate	d-Galactose	C00124	↑
1.2.2.4	Glucuronate pathway, Uronate pathway	M00014		d-Xylulose 5-Phosphate	C00231	↑
1.2.2.5	Nucleotide sugar biosynthesis	M00549	Glucose → UDP-Glucose	Alpha-d-Glucose	C00267	↑
1.2.2.6	Trehalose biosynthesis	M00565	D-Glucose 1-Phosphate → Trehalose	alpha,alpha-Trehalose	C01083	↑
1.2.3	*Central carbohydrate metabolism*					
1.2.3.1	Glycolysis (Embden–Meyerhof pathway)	M00001	Glucose → Pyruvate	Glycerone Phosphate	C00111	↑
1.2.3.2	Glycolysis, core module involving three-carbon compounds	M00002		Glycerone Phosphate	C00111	↑
1.2.3.3	Gluconeogenesis	M00003	Oxaloacetate → Fructose 6-Phosphate	Glycerone Phosphate	C00111	↑
1.2.3.4	Pentose phosphate pathway (pentose phosphate cycle)	M00004		d-Ribose 5-Phosphate	C00117	↑
1.2.3.5	PRPP biosynthesis	M00005	Ribose 5-Phosphate → PRPP	d-Ribose 5-Phosphate	C00117	↑
1.2.3.6	Pentose phosphate pathway, non-oxidative phase	M00007	Fructose 6-Phosphate → Ribose 5-Phosphate	d-Ribose 5-Phosphate	C00117	↑
1.2.3.7	Pentose phosphate pathway, archaea	M00580	Fructose 6-Phosphate → Ribose 5-Phosphate	d-Ribose 5-Phosphate	C00117	↑
1.2.3.8	Pentose phosphate pathway (pentose phosphate cycle)	M00004		d-Xylulose 5-Phosphate	C00231	↑
1.2.3.9	Pentose phosphate pathway, non-oxidative phase	M00007	Fructose 6-Phosphate → Ribose 5-Phosphate	d-Xylulose 5-Phosphate	C00231	↑
1.2.3.10	Glycolysis (Embden–Meyerhof pathway)	M00001	Glucose → Pyruvate	Alpha-d-Glucose	C00267	↑
1.2.4	*Lipopolysaccharide metabolism*					
1.2.4.1	CMP-KDO biosynthesis	M00063		d-Arabinose 5-Phosphate	C01112	↑
1.3	*Energy metabolism*					
1.3.1	*Carbon fixation*					
1.3.1.1	Reductive pentose phosphate cycle (Calvin cycle)	M00165		d-Ribose 5-Phosphate	C00117	↑
1.3.1.2	Reductive pentose phosphate cycle	M00167	Glyceraldehyde 3-Phosphate → Ribulose 5-Phosphate	d-Ribose 5-Phosphate	C00117	↑
1.4	*Secondary metabolism*					
1.4.1	*Biosynthesis of secondary metabolites*					
1.4.1.1	Monolignol biosynthesis	M00039	Phenylalanine/Tyrosine → Monolignol	Coniferyl Aldehyde	C02666	↓
1.4.1.2	Monolignol biosynthesis	M00039	Phenylalanine/Tyrosine → Monolignol	Sinapoyl Aldehyde	C05610	↓
1.5	*Others*					
1.5.1	Amino acid			l-Asparagine	C00152	↓
1.5.2	Oligosaccharides			Maltose	C00208	↑
1.5.3	Glycolysis/Gluconeogenesis			Beta-d-Glucose	C00221	↑
1.5.4	Arginine and proline metabolism			4-Aminobutyraldehyde	C00555	↑
1.5.5	Cysteine and methionine metabolism			O-Acetyl-l-Homoserine	C01077	↑
1.5.6	Lipopolysaccharide biosynthesis			d-Arabinose 5-Phosphate	C01112	↑
1.5.7	Biosynthesis of secondary metabolites			1-Aminocyclopropane-1-Carboxylate	C01234	↑
1.5.8	Biosynthesis of phenylpropanoids			Taxifolin	C01617	↓
1.5.9	Ascorbate and aldarate metabolism			l-Galactose	C01825	↑
1.5.10	Amino sugar and nucleotide sugar metabolism			Beta-l-Arabinose 1-Phosphate	C03906	↑
1.5.11	Biosynthesis of secondary metabolites			(2S)-2-Isopropyl-3-Oxosuccinate	C04236	↑
1.5.12	Biosynthesis of secondary metabolites			Pelargonidin 3-O-Glucoside	C12137	↓
2	Biosynthesis of secondary metabolites					
2.1	*Nucleotide and amino acid metabolism*					
2.1.1	*Cysteine and methionine metabolism*					
2.1.1.1	Cysteine biosynthesis	M00021	Serine → Cysteine	l-Serine	C00065	↓
2.1.1.2	Ethylene biosynthesis	M00368	Methionine → Ethylene	1-Aminocyclopropane-1-Carboxylate	C01234	↑
2.1.2	*Cofactor and vitamin biosynthesis*					
2.1.2.1	Pantothenate biosynthesis	M00119	Valine/l-Aspartate → Pantothenate	2-Dehydropantoate	C00966	↑
2.1.2.2	Ascorbate biosynthesis	M00114	Glucose 6-Phosphate → Ascorbate	l-Galactose	C01825	↑
2.1.2.3	Riboflavin biosynthesis	M00125	GTP → Riboflavin/FMN/FAD	6,7-Dimethyl-8-(d-Ribityl)Lumazine	C04332	↑
2.1.3	*Branched-chain amino acid metabolism*					
2.1.3.1	Valine/Isoleucine biosynthesis	M00019	Pyruvate → Valine/2-Oxobutanoate → Isoleucine	(S)−2-Aceto-2-Hydroxybutanoate	C06006	↑
2.2	*Carbohydrate and lipid metabolism*					
2.2.1	*Other carbohydrate metabolism*					
2.2.1.1	Photorespiration	M00532		l-Serine	C00065	↓
2.2.1.2	Trehalose biosynthesis	M00565	D-Glucose 1-Phosphate → Trehalose	alpha,alpha-Trehalose	C01083	↑
2.3	*Secondary metabolism*					
2.3.1	*Biosynthesis of secondary metabolites*					
2.3.1.1	Monolignol biosynthesis	M00039	Phenylalanine/Tyrosine → Monolignol	Coniferyl Aldehyde	C02666	↓
2.3.1.2	Monolignol biosynthesis	M00039	Phenylalanine/Tyrosine → Monolignol	Sinapoyl Aldehyde	C05610	↓
2.4	*Others*					
2.4.1	Flavonoid biosynthesis			Eriodictyol	C05631	↓
2.4.2	Flavonoid biosynthesis			Leucodelphinidin	C05909	↓
2.4.3	Biosynthesis of phenylpropanoids			Umbelliferone	C09315	↓
2.4.4	Biosynthesis of secondary metabolites			Pelargonidin 3-O-Glucoside	C12137	↓
2.4.5	Biosynthesis of phenylpropanoids			Taxifolin	C01617	↓
2.4.6	Biosynthesis of secondary metabolites			2-Isopropylmaleate	C02631	↑
2.4.7	Biosynthesis of secondary metabolites			(2S)-2-Isopropyl-3-Oxosuccinate	C04236	↑
3	Biosynthesis of amino acids					
3.1	*Nucleotide and amino acid metabolism*					
3.1.1	*Cysteine and methionine metabolism*					
3.1.1.1	Cysteine biosynthesis	M00609	Methionine → Cysteine	S-Adenosyl-l-Homocysteine	C00021	↓
3.1.1.2	Cysteine biosynthesis	M00021	Serine → Cysteine	l-Serine	C00065	↓
3.1.1.3	Cysteine biosynthesis	M00338	Homocysteine + Serine → Cysteine	l-Serine	C00065	↓
3.1.2	*Serine and threonine metabolism*					
3.1.2.1	Serine biosynthesis	M00020	Glycerate 3-Phosphate → Serine	l-Serine	C00065	↓
3.1.3	*Arginine and proline metabolism*					
3.1.3.1	Urea cycle	M00029		l-Citrulline	C00327	↓
3.1.3.2	Arginine biosynthesis	M00844	Ornithine → Arginine	l-Citrulline	C00327	↓
3.1.3.3	Arginine biosynthesis	M00845	Glutamate → Acetylcitrulline → Arginine	l-Citrulline	C00327	↓
3.1.4	*Branched-chain amino acid metabolism*					
3.1.4.1	Valine/Isoleucine biosynthesis	M00019	Pyruvate → Valine/2-Oxobutanoate → Isoleucine	(S)-2-Aceto-2-Hydroxybutanoate	C06006	↑
3.1.4.2	Isoleucine biosynthesis	M00570	Threonine → 2-Oxobutanoate → Isoleucine	(S)-2-Aceto-2-Hydroxybutanoate	C06006	↑
3.2	*Carbohydrate and lipid metabolism*					
3.2.1	*Central carbohydrate metabolism*					
3.2.1.1	Glycolysis, core module involving three-carbon compounds	M00002		Glycerone Phosphate	C00111	↑
3.2.1.2	PRPP biosynthesis	M00005	Ribose 5-Phosphate → PRPP	d-Ribose 5-Phosphate	C00117	↑
3.2.1.3	Pentose phosphate pathway, non-oxidative phase	M00007	Fructose 6-Phosphate → Ribose 5-Phosphate	d-Ribose 5-Phosphate	C00117	↑
3.2.1.4	Pentose phosphate pathway, archaea	M00580	Fructose 6-Phosphate → Ribose 5-Phosphate	d-Ribose 5-Phosphate	C00117	↑
3.2.1.5	Pentose phosphate pathway, non-oxidative phase	M00007	Fructose 6-Phosphate → Ribose 5-Phosphate	d-Xylulose 5-Phosphate	C00231	↑
3.3	*Others*					
3.3.1	Amino acid			l-Asparagine	C00152	↓
4	Carbon metabolism					
4.1	*Nucleotide and amino acid metabolism*					
4.1.1	*Serine and threonine metabolism*					
4.1.1.1	Serine biosynthesis	M00020	Glycerate 3-Phosphate → Serine	l-Serine	C00065	↓
4.1.2	Cysteine and methionine metabolism					
4.1.2.1	Cysteine biosynthesis	M00021	Serine → Cysteine	l-Serine	C00065	↓
4.2	*Energy metabolism*					
4.2.1	*Methane metabolism*					
4.2.1.1	Formaldehyde assimilation, serine pathway	M00346		l-Serine	C00065	↓
4.2.1.2	Formaldehyde assimilation, xylulose monophosphate pathway	M00344		Glycerone Phosphate	C00111	↑
4.2.1.3	Formaldehyde assimilation, ribulose monophosphate pathway	M00345		Glycerone Phosphate	C00111	↑
4.2.2	*Carbon fixation*					
4.2.2.1	Reductive pentose phosphate cycle (Calvin cycle)	M00165		d-Ribose 5-Phosphate	C00117	↑
4.2.2.2	Reductive pentose phosphate cycle	M00167	Glyceraldehyde 3-Phosphate → Ribulose 5-Phosphate	d-Ribose 5-Phosphate	C00117	↑
4.3	*Carbohydrate and lipid metabolism*					
4.3.1	*Central carbohydrate metabolism*					
4.3.1.1	Glycolysis (Embden-Meyerhof pathway)	M00001	Glucose → Pyruvate	Glycerone Phosphate	C00111	↑
4.3.1.2	Glycolysis, core module involving three-carbon compounds	M00002		Glycerone Phosphate	C00111	↑
4.3.1.3	Pentose phosphate pathway (Pentose phosphate cycle)	M00004		d-Ribose 5-Phosphate	C00117	↑
4.3.1.4	PRPP biosynthesis	M00005	Ribose 5-Phosphate → PRPP	d-Ribose 5-Phosphate	C00117	↑
4.3.1.5	Pentose phosphate pathway, non-oxidative phase	M00007	Fructose 6-Phosphate → Ribose 5-Phosphate	d-Ribose 5-Phosphate	C00117	↑
4.3.1.6	Pentose phosphate pathway, archaea	M00580	Fructose 6-Phosphate → Ribose 5-Phosphate	d-Ribose 5-Phosphate	C00117	↑
4.3.1.7	Pentose phosphate pathway (Pentose phosphate cycle)	M00004		d-Xylulose 5-Phosphate	C00231	↑
4.3.1.8	Pentose phosphate pathway, non-oxidative phase	M00007	Fructose 6-Phosphate → Ribose 5-Phosphate	d-Xylulose 5-Phosphate	C00231	↑
4.3.1.9	Glycolysis (Embden-Meyerhof pathway)	M00001	Glucose → Pyruvate	Alpha-d-Glucose	C00267	↑
4.3.2	*Other carbohydrate metabolism*					
4.3.2.1	Photorespiration	M00532		l-Serine	C00065	↓
4.4	*Others*					
4.4.1	Glycolysis/Gluconeogenesis			Beta-d-Glucose	C00221	↑
5	Galactose metabolism					
5.1	Glycolysis, core module involving three-carbon compounds			Glycerone Phosphate	C00111	↑
5.2	Galactose degradation			d-Galactose	C00124	↑
5.3	Inositol phosphate metabolism			Myo-Inositol	C00137	↑
5.4	Glycolysis (Embden–Meyerhof pathway)			Alpha-d-Glucose	C00267	↑
5.5	Galactose metabolism			Galactinol	C01235	↑
6	ABC transporters					
6.1	Phosphate and amino acid transporters			l-Serine	C00065	↓
6.2	Monosaccharide transporters			Myo-Inositol	C00137	↑
6.3	Oligosaccharide, polyol, and lipid transporters			Maltose	C00208	↑
6.4	Oligosaccharide, polyol, and lipid transporters			alpha,alpha-trehalose	C01083	↑
7	Cysteine and methionine metabolism					
7.1	Methionine degradation	M00035		S-Adenosyl-l-Homocysteine	C00021	↓
7.2	Glycine, serine and threonine metabolism			l-Serine	C00065	↓
7.3	Aspartate metabolism			O-Acetyl-l-Homoserine	C01077	↑
7.4	Propanoate metabolism			1-Aminocyclopropane-1-Carboxylate	C01234	↑
8	Flavonoid biosynthesis					
8.1	Flavone and flavonol biosynthesis			Vitexin	C01460	↓
8.2	Flavone and flavonol biosynthesis			Taxifolin	C01617	↓
8.3	Flavanone biosynthesis			Eriodictyol	C05631	↓
8.4	Flavan 3,4-diols biosynthesis			Leucodelphinidin	C05909	↓
9	Glycerophospholipid metabolism					
9.1	Ether lipid metabolism			Glycerone Phosphate	C00111	↑
9.2	Phosphocholine biosynthesis			N-Methylethanolamine Phosphate	C01210	↑
9.3	Phosphocholine biosynthesis			Phosphodimethylethanolamine	C13482	↑
10	Glycolysis/gluconeogenesis					
10.1	Core module involving three-carbon compounds			Glycerone Phosphate	C00111	↑
10.2	Starch and sucrose metabolism			Beta-d-Glucose	C00221	↑
10.3	Starch and sucrose metabolism			Alpha-d-Glucose	C00267	↑
11	Amino sugar and nucleotide sugar metabolism					
11.1	Uridine diphosphate sugar metabolism			Alpha-d-Glucose	C00267	↑
11.2	Uridine diphosphate sugar metabolism			Beta-d-Fructose	C02336	↑
11.3	Uridine diphosphate sugar metabolism			Beta-l-Arabinose 1-Phosphate	C03906	↑
12	Carbon fixation in photosynthetic organisms					
12.1	Glycolysis			Glycerone Phosphate	C00111	↑
12.2	Reductive pentose phosphate cycle			d-Ribose 5-Phosphate	C00117	↑
12.3	Reductive pentose phosphate cycle			d-Xylulose 5-Phosphate	C00231	↑
13	2-Oxocarboxylic acid metabolism					
13.1	Pyruvate reductive amination			2-Isopropylmaleate	C02631	↑
13.2	Pyruvate reductive amination			(2S)−2-Isopropyl-3-Oxosuccinate	C04236	↑
13.3	Pyruvate reductive amination			(S)−2-Aceto-2-Hydroxybutanoate	C06006	↑
14	Phenylpropanoid biosynthesis					
14.1	Sinapate derivatives			1-O-Sinapoyl-Beta-d-Glucose	C01175	↓
14.2	Coniferyl alcohol derivatives			Coniferyl Aldehyde	C02666	↓
14.3	Sinapate derivatives			Sinapoyl Aldehyde	C05610	↓
15	Pentose phosphate pathway					
15.1	PRPP biosynthesis		Ribose 5-Phosphate → PRPP	d-Ribose 5-Phosphate	C00117	↑
15.2	PRPP biosynthesis			Beta-d-Glucose	C00221	↑
15.3	PRPP biosynthesis			d-Xylulose 5-Phosphate	C00231	↑
16	Anthocyanin biosynthesis					
16.1	Flavonoids			Chrysanthemin	C08604	↓
16.2	Flavonoids			Pelargonidin 3-O-Glucoside	C12137	↓
16.3	Flavonoids			Cyanidin 5-O-Glucoside	C16298	↓
17	Ascorbate and aldarate metabolism					
17.1	Sugar alcohols			Myo-Inositol	C00137	↑
17.2	Ascorbate degradation		Ascorbate → d-Xylulose 5-Phosphate	d-Xylulose 5-Phosphate	C00231	↑
17.3	Aldoses			l-Galactose	C01825	↑
18	Valine, leucine and isoleucine biosynthesis					
18.1	Pyruvate metabolism			2-Isopropylmaleate	C02631	↑
18.2	Leucine biosynthesis			(2S)-2-Isopropyl-3-Oxosuccinate	C04236	↑
18.3	Isoleucine biosynthesis			(S)-2-Aceto-2-Hydroxybutanoate	C06006	↑
19	Glycine, serine and threonine metabolism					
19.1	Pyruvate, cysteine and tryptophan metabolism			l-Serine	C00065	↓
20	Pentose and glucuronate interconversions					
20.1	Glucuronate interconversion			Glycerone Phosphate	C00111	↑
20.2	Pentose interconversion			d-Xylulose 5-Phosphate	C00231	↑
21	Fructose and mannose metabolism					
21.1	Core module involving three-carbon compounds			Glycerone Phosphate	C00111	↑
21.2	Fructose biosynthesis			Alpha-d-Glucose	C00267	↑
22	Starch and sucrose metabolism					
22.1	Oligosaccharides			Maltose	C00208	↑
22.2	Oligosaccharides			alpha,alpha-Trehalose	C01083	↑
23	Inositol phosphate metabolism					
23.1	Core module involving three-carbon compounds			Glycerone Phosphate	C00111	↑
23.2	Inositol phosphate metabolism		Ins(1,3,4,5)P4 → Ins(1,3,4)P3 → Myo-Inositol	Myo-Inositol	C00137	↑
24	Flavone and flavonol biosynthesis					
24.1	Flavones			Vitexin	C01460	↓
24.2	Flavones			Isovitexin	C01714	↓
25	Phenylalanine, tyrosine and tryptophan biosynthesis					
25.1	Shikimate pathway			Quinate	C00296	↑

KEGG pathways and modules that involve compounds identified in Table 1 were generated by KEGG Mapper^75^; Bin codes denote the hierarchical structure of KEGG pathways and modules used to generate MapMan^77^ style representation of the identified compounds (Fig. 4); ↑ refers to a compound with positive *t* stat value (high- > low-sugar group), whereas ↓ refers to a compound with a  negative *t* stat value (high- < low-sugar group).

All major pathways, i.e., metabolic, biosynthesis of secondary metabolites, biosynthesis of amino acids and carbon metabolism, were broadly classified into nucleotide and amino acid, carbohydrate and lipid and energy metabolism modules (Fig. 5). Other modules related to primary metabolism comprised galactose metabolism, glycolysis, amino sugar metabolism, carbon fixation, oxocarboxylic acid metabolism, pentose phosphate pathway, ascorbate metabolism, pentose and glucuronate conversions, fructose and mannose metabolism, starch and sucrose metabolism, inositol phosphate metabolism and glycerophospholipid metabolism, while modules related to secondary metabolism comprised flavone, flavonol, anthocyanin, flavonoid and phenylpropanoid biosynthesis (Table 2; Fig. 5).

In all major pathways, compounds related to metabolism of the amino acids cysteine, methionine, serine, threonine, arginine and proline were found in low concentrations in the high-sugar group. However, their intermediate products (S)-2-Aceto-2-Hydroxybutanoate, O-Acetyl-l-Homoserine, (2S)-2-Isopropyl-3-Oxosuccinate and 2-Dehydropantoate were at higher concentrations (Fig. 5). These intermediate products were in turn involved in the biosynthesis of branched-chain amino acids leucine, isoleucine and valine (M00019, M00570 and M00432; Fig. 5; Table 2). On the other hand, compounds related to carbohydrate, lipid and energy metabolism were at high concentrations, while those related to the biosynthesis of secondary metabolites were low (Fig. 5). L-Serine and S-Adenosyl-l-Homocysteine were primarily involved in cysteine, methionine, serine and threonine metabolism (Modules M00035, M00609, M00021, M00338 and M00020; Table 2). l-Citrulline was involved in arginine and proline metabolism via the urea cycle (M00029), and (S)-2-Aceto-2-Hydroxybutanoate was involved in branched chain amino acid metabolism (M00019 and M00570). Glycerone phosphate, a breakdown product of fructose 1, 6-Biphosphate and an isomer of 3-Phosphoglyceraldehyde (3-PGA), d-Ribose 5-Phosphate, d-Xylulose 5-Phosphate and alpha-d-Glucose, all found in higher concentrations in the high-sugar group, were involved in the central carbohydrate metabolism via glycolysis/gluconeogenesis (M00001, M00002 and M00003) and the pentose phosphate pathway (M00007, M00580 and M00004; Fig. 5; Table 2). Other compounds such as myo-Inositol were involved in lipid metabolism via the inositol phosphate metabolism module (M00131: Table 2), and d-Arabinose 5-Phosphate was involved in lipopolysaccharide biosynthesis (M00063: Table 2), both reportedly at increased concentrations in the high-sugar group (Fig. 5).

l-Serine (low concentration), d-Galactose, d-Xylulose 5-Phosphate, alpha-d-Glucose and alpha,alpha-Trehalose (all high concentrations) were some of the metabolites involved in other carbohydrate metabolism modules which comprised photorespiration (M00532), galactose degradation (M00632), glucuronate pathway (M00014) and nucleotide sugar biosynthesis (M00549). Ryegrass being a C-3 plant^30^, the energy metabolism pathway comprising carbon fixation and methane metabolism modules involved d-Ribose 5-Phosphate (high concentration) in the Calvin cycle (M00165) and glycerone phosphate (high concentration) in formaldehyde assimilation (M00344 and M00345), respectively. Biosynthesis of secondary metabolites involved coniferyl aldehyde, sinapoyl aldehyde, 1-O-Sinapoyl-beta-d-Glucose and 1-O-(4-Coumaroyl)-beta-d-Glucose, all in low concentrations, and related to the biosynthesis of monolignols (M00039). Compounds classified as others’ represented metabolites that did not feature in any modules, but were indirectly involved in the corresponding pathways. In that light, compounds classified as others’ in metabolic pathways mainly comprised amino acids, sugars and sugar phosphates (predominantly in higher concentrations), and those classified under the biosynthesis of secondary metabolites comprised umbelliferone, vitexin, isovitexin, orientin, isoorientin, pelargonidin-3-O-beta-d-Glucoside, cyanidin-3-O-beta-d-Glucoside and cyanidin 5-O-beta-d-Glucoside, (predominantly in lower concentrations; Fig. 5; Table 2).

Compounds belonging to minor pathways were classified as those involved in primary or secondary metabolism (Fig. 5). Clearly, compounds related to the primary metabolism of sugars via galactose metabolism, glycolysis, amino sugar metabolism, carbon fixation, oxocarboxylic acid metabolism, pentose phosphate pathway, ascorbate metabolism, pentose and glucuronate conversions, fructose and mannose metabolism, starch and sucrose metabolism, inositol phosphate metabolism and lipids via glycerophospholipid metabolism, were all found at higher concentrations in high-sugar grasses. Likewise, compounds related to secondary metabolism via flavone, flavonol, anthocyanin, flavonoid, and phenylpropanoid biosynthesis, were all found in lower concentrations (Fig. 5; Table 2). Amongst the ABC transporters, l-Serine, a phosphate and amino acid transporter was found in low concentration, whereas myo-Inositol, maltose and alpha, alpha-Trehalose, mono- and oligosaccharide transporters, were found at higher concentrations (Fig. 5; Table 2). Quinate/Quinic acid, involved in the biosynthesis of phenylalanine, tyrosine and tryptophan via the shikimate pathway, was found at higher concentration in high-sugar grasses, and so were compounds involved in the biosynthesis of the branched chain amino acids valine, leucine and isoleucine (Fig. 5; Table 2).

Overall, a snapshot of metabolic pathways in high-sugar grasses revealed: an increase in concentrations of compounds involved in primary metabolism, mainly comprising sugars; decrease in concentrations of compounds involved in secondary metabolism, mainly comprising flavonoids and lignins; decrease in concentrations of compounds involved in cysteine, methionine, serine, arginine, proline and threonine metabolism, and; a concomitant increase in concentrations of compounds involved in the metabolism of branched chain amino acids valine, isoleucine and leucine, and aromatic amino acids phenylalanine, tyrosine and tryptophan.

## Discussion

The distribution of 715 ryegrass genotypes based on their total sugar content (Fig. 1), provides an overview of their performance under natural NZ autumn conditions. High-sugar content in leaves has a direct relationship with the metabolisable energy of ryegrass, which in turn enhances protein capture and supply to the ruminant^16,31^. While, seasonal variation of fructan content has also been hypothesised^32^, we have only measured fructan content at a single seasonal time point. A time-resolved analysis of fructan diversity is therefore expected to shed more light on the genotype and/or genotype × environment interactions^33^ in the high-sugar group identified here (Supplementary Table 1).

We also hypothesised that the DP contributes to differences in total sugar content, both between and within the high- and low-sugar groups. Between the high- and low-sugar groups, all three DP classes (low, mid or high) were significantly higher in the high-sugar group (Fig. 2a). However, as reported earlier^15^, within the high-sugar group, the contribution of high-DP fructans to the total fructan content was greater than the low-DP fructans (Fig. 2a). For the standard high-sugar genotypes (Aber cultivars), high-DP fructans are considered critical in retaining the total sugar content under seasonal variation compared to normal genotypes^34^. Therefore, considering high-DP fructans are sought for breeding reliable high-sugar genotypes, 11 genotypes which have greater high-DP content than Aberdart, have been identified (Fig. 2c). Given that fructan accumulation and degradation in ryegrass is still poorly understood^14^, the negative correlation between high- and low-DPs within the high-sugar group (Fig. 2b), remains unexplained and deems further investigation. The metabolomics approach employed here for fructan analysis has therefore led to a better understanding of fructan accumulation in high-sugar genotypes prior to genotype selection. Moreover, the contrast provided here with the low-sugar group, in the context of lipids, polar and semi-polar compounds (Figs. 3 and 4), has established a platform to scrutinise underlying biological mechanisms (Fig. 5; Table 2).

Another route to high-metabolisable energy has been via increased lipid content in leaves^35^. Elevated levels of triglycerides/triacylglycerols and poly-unsaturated fatty acids at the cost of proteins, would lead to improved nitrogen utilisation and feed conversion efficiency of the ruminants^35^, resulting in enhanced nutritional value of the end-products^36^. Exclusive efforts aimed at high-lipid content, primarily based on metabolic engineering, are therefore in practice^35,37,38^. While fatty acids have been the focus of this endeavour^36^, other lipid classes or the lipidome have largely been ignored. The lipid profile presented here (Fig. 4a), therefore potentially marks the first comprehensive report of the ryegrass lipidome (Supplementary Data 2). A negative relationship between sugar and fatty acid content (Fig. 4b) has also been reported by Morgan^36^. When the sugar content is low due to diurnal, environmental or genotypic factors, plants have the ability to redirect cellular activity towards lipid catabolism, resulting in an increase in fatty acids^39^. Manipulating this carbon flux towards lipid biosynthesis has also been suggested to achieve elevated levels of lipids in plants^37^.

A few classes of phospholipids, i.e., phosphatidylserine, phosphatidylglycerol and phosphatiylcholine, diglycerides and galactolipids were found in higher concentrations in the low-sugar group and triglycerides were higher in the high-sugar group (Fig. 4a). Galactolipids are found in thylakoid membranes of chloroplasts and are abundant in photosynthetically active leaves^40^. A hypothesis that an increase in FA content correlates with chlorophyll content in leaves, and thereby galactolipids, was tested by Morgan^36^. In general, polar lipids mainly contributed by galactolipids had a positive correlation with FA content, whereas neutral lipids mainly comprising triglycerides had a negative correlation^36^. These results are in accordance with the present study (Fig. 4). Triglycerides are storage lipids and, until recently, were thought to be absent in leaves^41^. They are believed to be intermediate products in the catabolism of galactolipids to sucrose, mostly accumulating in leaves during the day^41^. While their role in lipid metabolism is largely unknown, their elevated levels in high-sugar grasses provides sufficient context to pursue this result further.

Few studies have investigated the impact of changes in primary metabolism on the fluxes into secondary metabolism^42,43^. Non-targeted metabolomics, through a wide coverage of primary and secondary metabolites as demonstrated in this study, is well positioned to elucidate global metabolic changes, and thereby the cross-linkages between primary and secondary metabolism. Combined with other –omics data, metabolomics provides a robust platform for biological interpretation. In spite of these inherent advantages, non-targeted studies are limited by the bottleneck of compound identification, resulting in compounds predominantly identified with low to medium-level confidence^44^. Taking cues from non-targeted studies and proceeding towards unequivocal identification using targeted approaches is therefore recommended. Here, a snapshot of metabolic activity in the high-sugar group (Fig. 5) revealed an increase in concentrations of compounds involved in primary metabolism, mainly comprising sugars, a decrease in concentrations of compounds related to metabolism of the amino acids cysteine, methionine, serine, threonine, arginine and proline, increase in concentrations of compounds involved in the biosynthesis of aromatic and branched chain amino acids, and a concomitant decrease in compounds involved in secondary metabolism, mainly comprising flavonoids and lignins.

Plant secondary metabolites are of interest due to their bewildering diversity, and their roles in defence, stress response, UV protection, allelopathy and signalling^45^. The link between carbohydrate and secondary metabolism is primarily mediated by the shikimate pathway, through synthesis of aromatic amino acids, leading into the phenylpropanoid pathway^46,47^. Quinic acid, a constituent of the phenylpropanoid pathway, is a precursor of chlorogenic acid^47^, and also involved in the biosynthesis of lignins and flavonols. Quinic acid was found at higher concentrations in the high-sugar group, and on the other hand, coniferyl aldehyde and sinapoyl aldehyde (lignin subunits^48^), were found in lower concentrations (Table 1). Chlorogenic acid concentrations were not significantly different between the high- and low-sugar groups. In sorghum (*Sorghum bicolor*) plants with high biomass, quinic acid and lignin contents were high, whereas in the high-sugar ryegrass cultivar ‘Aberdove’, the concentration of chlorogenic acid was high^49^. A partitioning of quinic acid towards biosynthesis of either lignin or chlorogenic acid was therefore hypothesised^47^. However, the preferential flux towards either of these compounds is unknown. Grass-fibre composition is another key target of breeding which affects feed intake and digestibility^50^. This is largely determined by cell wall components, and lignins, complex polyphenolic polymers and end products of the phenylpropanoid pathway are amongst the major cell wall constituents^51^. The shikimate pathway described here in the context of total sugar content, should persuade further studies on secondary metabolism in ryegrass. In addition to the shikimate pathway, the interface between primary and secondary metabolism in plants is far more complex, with the production of secondary metabolites associated with glycolysis, TCA cycle, aliphatic amino acids, pentose phosphate pathway^52^ and nitrogen status^53^.

Towards the primary objective of screening for high-sugar cultivars, we set-off with a survey of 715 ryegrass genotypes based on their fructan content. However, breeding for select traits requires a thorough understanding of the population wide diversity of these traits, and their genetic control mechanisms^21^. Non-targeted metabolomics studies, as employed here, delivered the broadest coverage of metabolites, thereby enabling a better understanding of the underlying biochemical mechanisms. A snapshot of metabolites and the corresponding modules/pathways they represent in the high-sugar group (Fig. 5), established key insights on primary and secondary metabolism, that merit further investigation. This study therefore signifies one of many avenues that can be explored with these data. For example, the low-sugar group indirectly led to genotypes with greater lipid content, thereby creating opportunities to tap into lipid content through breeding applications. Likewise, the flux from primary to secondary metabolism, as reported here, may facilitate breeding strategies specifically targeting select secondary metabolites. Studies exploring these avenues in secondary metabolites are already underway^20,54^. In conclusion, we have established levers that help explain biochemical activity in ryegrass, which when operated towards specific objectives can deliver desired traits. The raw files from this study, maintained at MetaboLights database, we envisage will cater to further explorations towards these objectives.

## Methods

### Experimental

*Plant material*: Ryegrass seeds were obtained from the Margot Forde Germplasm Centre at AgResearch Limited, Grasslands Research Centre, Palmerston North, New Zealand. Seeds of 724 genotypes (denoted by a specific code, e.g., PG0698) which included NZ cultivars (82), natural population/ecotypes (49), NZ elite breeding populations (336), overseas cultivars (181), enhanced germplasm (71) and unknown cultivars (5) were selected. Five clonal replicates (*n* = 5; denoted as PG0698_1 for the first replicate) per genotype were cultivated and used for metabolomics studies. Plant trial conditions are described in Supplementary Methods.

Perennial ryegrass forms a natural symbiotic association with epichloae fungi (*Epichloë spp*.)^50^, which produce metabolites that protect the host from biotic and abiotic stresses^55^. It has been shown that infection with these endophytes affects the metabolic profiles of ryegrass^49,56^. To ensure that the ryegrass metabolome alone was analysed and reported, endophyte-free plants were used. This was achieved by heat and fungicide treatment of seed to kill the fungus, prior to germinating and growing the plants. After growing out and prior to clonal replication, the endophyte-free status of plants was assessed by an immunoblot analysis^57^ of 10 tillers per plant. Subsequently, the presence of peramine, a pyrrolopyrazine alkaloid^58^ specific to most endophytes, was also monitored. Nine genotypes that showed presence of peramine were identified and excluded (Supplementary Figure 9). This resulted in 715 genotypes from 118 populations for subsequent analyses. Here, a genotype refers to a group of plants with the same genetic makeup, while a population consists of one or more genotypes grouped together based on a common trait (genotypic/phenotypic/geographical origin). For example, PG0698 refers to the genotype in a population named ‘Hillary’, a NZ based cultivar (Supplementary Table 1).

*Extractions*: At 60 days after transplanting, leaves were harvested during late autumn (May 2012), snap-frozen in liquid nitrogen, freeze-dried, ground to a coarse powder, transferred to glass vials and stored at −80 °C until further use. Five aliquots of 50 mg each (4 for analyses and 1 standby) of each sample were weighed into microcentrifuge tubes. A surrogate QC sample^59^ comprising random amounts of ground ryegrass leaf material, irrespective of the presence or absence of endophyte was used. Since this QC sample was not representative of the sample set, it was solely used for monitoring sample degradation and for tracking run-order effects within a batch and not used for batch normalisations or ensuing data processing.

*Polar and semi-polar compounds*. A single aliquot (50 mg) was used for analysing both polar and semi-polar compounds. One millilitre (1 ml) of extraction solvent comprising acetonitrile:water containing 0.1% formic acid (50:50, v/v), and 2′,7′-Dichlorofluorescein (1 µg/ml; CAS No. 76-54-0; MW = 401.2) and l-Tyrosine-3, 3-d_2_ (10 µg/ml; CAS No. 72963-72-0; MW = 183.20) as internal standards for semi-polar and polar compounds respectively, were added to the aliquot. A ceramic bead was added and samples were mixed in a bead-mill homogeniser (TissueLyser II; Qiagen, Valencia, CA, USA) for 3 min, after which they were centrifuged for 6 min (18188*g*). Approximately 500 µl of the aqueous extract was transferred to an autosampler vial and stored at −20 °C until analysis.

*Lipids.* For lipids, all procedures were similar to that of polar and semi-polar compounds, except that the extraction solvent was made of damp chloroform:methanol (67:33, v/v), containing 2′,7′-Dichlorofluorescein (20 µg/ml) as an internal standard.

*Fructans*. Boiling water (1.5 ml) was added to another aliquot (50 mg). After homogenisation, samples were placed in a hot water bath (90 °C) for 30 min. Samples were cooled to room temperature, centrifuged, and approximately 500 µl of the aqueous extract was transferred to an autosampler vial for analysis. Samples were stored (−20 °C) for a minimal period to avoid fructan degradation.

*Fatty acid methyl esters (FAMEs)*. After extraction of fructans, the remaining supernatant was discarded and the pellet was freeze-dried overnight. The dried aliquot was transferred to a 15 ml Falcon tube containing 50 µl of pentadecanoic acid (C15:0; CAS No. 1002-84-2; MW = 242.4) in heptane (4 mg/ml) as an internal standard. After the addition of 1 ml of 1 M methanolic HCl reagent^60^, the tubes were purged with nitrogen, sealed and heated in a hot water bath (80 °C) for 1 h. Tubes were then cooled to room temperature, and 50 µl of heptadecanoic acid methyl ester (Premethyl ester C17:0; CAS No. 1731-92-6; MW = 284.48) in heptane (4 mg/ml) was added as another internal standard. A total of 600 µl of heptane and 1 ml of 0.9% sodium chloride (w/v; NaCl) were added and the tubes were manually shaken to extract FAMEs into the heptane phase. Totally, 150 µl of the heptane layer was transferred to a 250 µl glass insert fitted in an autosampler vial for GC–MS analysis.

*Chromatography and tandem mass-spectrometry*: *(U)HPLC-MS.* (U)HPLC-MS conditions for analysis of polar and semi-polar compounds using ZIC-pHILIC^61^ and C18^62^ columns, were as described in respective references. An Exactive Orbitrap (Thermo Fisher Scientific, Waltham, MA, USA) mass spectrometer with electrospray ionisation was used for analyses in both positive and negative modes. Fructans were analysed as described by Harrison, et al.^63^ using a porous graphitised carbon column and LTQ linear ion trap mass spectrometer (Thermo Fisher Scientific, Waltham, MA, USA) with electrospray ionisation in negative mode.

The Thermo LC–MS system (Thermo Fisher Scientific, Waltham, MA, USA) for analysis of lipids consisted of an Accela 1250 quaternary UHPLC pump, a PAL auto-sampler fitted with a 15,000 psi injection valve (CTC Analytics AG., Zwingen, Switzerland) a 20 μl injection loop, and a Q-Exactive Orbitrap^TM^ mass spectrometer with electrospray ionisation. A C1 column (50 × 2.1 mm, 5 µm; Thermo Fisher Scientific, Waltham, MA, USA), maintained at 25 °C with a gradient elution programme and a flow rate of 500 µl/min, was used for chromatographic separation. The mobile phase comprised water containing 0.1% formic acid (Solvent A) and isopropanol:acetonitrile containing 0.1% formic acid (50:50, v/v; Solvent B). The gradient was set to hold solvent A at 80% from 0 to 1 mins, gradually decline to 0% at 18.1 mins, maintained at the same level up to 20 mins, increased to 80% at 20.1 mins and finally allowed to equilibrate as such for the rest of the programme, i.e., 25 min. The samples were cooled in the auto-sampler at 4 °C and the injection volume of each sample was 2 μl. The first 1.5 min and the last 5 min of the chromatogram were diverted to waste. Both full and data dependent MS^2^ scans were collected in profile data acquisition mode. For full scan mode, a mass resolution setting of 35,000 was set to record a mass range of *m*/*z* 200–2000 with a maximum trap fill time of 250 ms. For MS^2^ scan mode, the same mass resolution setting was maintained with a maximum trap fill time of 120 ms. The isolation window of selected MS^1^ scans was ± 1.5 *m/z* with a collision energy of 30 eV. Samples were run in both positive and negative ionisation modes separately. Positive-ion mode parameters were as follows: spray voltage, 4.0 kV; capillary temperature, 275 °C; capillary voltage, 90 V, tube lens 120 V. Negative-ion mode parameters were as follows: spray voltage, −4.0 kV; capillary temperature, 275 °C; capillary voltage, −90 V, tube lens −100 V. The nitrogen source gas desolvation settings were the same for both modes (arbitrary units): sheath gas, 40; auxiliary gas, 10; sweep gas, 5. The Xcalibur software package provided by the manufacturer was used to create these settings.

*GC–MS*. Analysis of FAMEs was undertaken using a Thermo DSQ II Trace Ultra gas chromatograph (Thermo Fisher Scientific, Waltham, MA, USA) fitted with a DB5 GC capillary column. GC–MS conditions were as described by Browse et al.^60^.

*Standards and reagents*: All standards were purchased from Sigma–Aldrich Chemicals Co. (St. Louis, MO). Ultrapure water was obtained from a Milli-Q system (Millipore, Bedford, MA). All solvents used were of Optima LC–MS grade and were purchased from Thermo Fisher Scientific (Auckland, New Zealand).

### Data analysis

*Sample sequence and batches*: For each stream of analysis, samples (3575) were systematically randomised across 36 batches, making sure that two clonal replicates of a genotype were not present in the same batch. A single batch comprised approximately 100 samples interspersed with a QC sample for every 10 samples (10–12 QC samples per batch). The sequence comprised blanks, QC and samples run in positive mode, followed by the same order in negative mode. Fructans and FAMEs were analysed in negative-^63^ and positive- ionisation modes alone, respectively.

*QC monitoring and troubleshooting*: For each batch, the quality of runs was determined by constantly monitoring the respective internal standard in QC samples for: (1) consistency in retention time (±0.5 min), (2) mass accuracy (±5 ppm) and (3) signal intensity. In the instance of constant drift in one of these parameters, the batch was immediately stopped and re-run after recalibrating the mass spectrometer. After completion of the run sequence, samples of each batch were retained at −20 °C until another QC check (based on PCA) was conducted. PCA of samples classified based on run-order within each batch was used to reveal any significant run-order effects. Where a significant run-order effect was apparent, the batches were re-run. Otherwise, batches were passed on to a super batch (a collection of batches that have passed the QC tests), for further processing. Score plots of PCA provide a simple and quick qualitative assessment of variability within a sample set^64^.

*Fructans*: Fructan identification and DP measurements were based on XCMS^65^ (Supplementary Table 2) and an in-house R script. Essentially, a target list from DP2 to DP20, denoting the parent mass, dimer and commonly formed adducts (formic acid and chlorine) for each DP, with corresponding retention times, was created based on respective extracted ion chromatograms. The script was used for (1) peak detection, (2) peak grouping, (3) retention time correction, (4) peak filling, (5) normalisation of run-order within each batch^20^, (6) normalisation of batch-effects using comBat^66^, (7) matching the resultant data matrix with the target list and (8) creating a table with peak intensities for each DP, including the molecular ion, dimer and adduct masses, across all samples.

Peak intensities of all ions representing each DP were summed, and the sum was multiplied by the number of hexose units, to establish a common baseline for comparisons. An exemplar is presented in the case of DP3 (Supplementary Table 3). As a measure of the total sugar content, fructans in the low (DP3, 4 and 5), mid (DP10, 11 and 12) and high (DP18, 19 and 20) DP ranges were added.

To delineate maximum separation between high- and low-sugar groups and maintain consistency of results, a two-tier criterion was established. First, the 715 genotypes (*n* = 5) were ranked as top 10% and bottom 10% based on total sugar content, and second, the full sample set (3575) was ranked as top 10% and bottom 10% based on total sugar content. Within the top 10% and bottom 10% of genotypes identified, only genotypes with three or more (out of five) clonal replicates were selected for comparisons. This two-tier criterion enabled identification of genotypes with minimum variation. Raw files corresponding to these high- (*n* = 133) and low-sugar (*n* = 106) samples from other analytical streams (polar, semi-polar, FAMEs and lipids) were collated for further comparative analysis.

*Lipids, polar and semi-polar compounds*: XCMS^65^ (Supplementary Table 2) and in-house R scripts with appropriate parameters for UHPLC (C18) and HPLC (Lipids and HILIC) settings were used for: (1) peak detection using centwave; (2) grouping; (3) retention time alignment using obiwarp; (4) peak re-grouping and (5) filling of missing peaks.

The resultant data matrices were cleaned and post-processed by using: (1) diffreport function of XCMS to generate extracted ion chromatograms of all identified peaks, and eliminating the ones that represented background noise, and; (2) CAMERA^67^ to identify and eliminate isotopes (HILIC and C18) and (3) normalisation for batch-effects using comBat^66^. The final data matrices of metabolic features were used for local library matching and statistical analysis. Metabolic features, defined here as molecular entities or ion types with a unique mass-to-charge ratio (*m/z*) and retention time.

*LipidSearch and local library matching*: Lipid identification was performed using LipidSearch software (Thermo Fisher Scientific, USA)^68^. Raw lipid files corresponding to high- and low-sugar groups were uploaded to LipidSearch separately for positive and negative ionisation modes. Product ion search on Q-Exactive^TM^ data was selected with a mass tolerance of 6 ppm for precursor ions and 10 ppm for product ions, along with a selection of lipid classes (Supplementary Table 4). Lipid species/classes identified for each file were then merged based on retention time alignments and a single file with the merged results, one each for each ionisation mode, was generated. This library of identified lipids was matched against the final data matrices based on parent mass and retention times. Peak intensities generated by XCMS settings for the identified lipids were ultimately used for further statistical analyses.

The lipidomics results shown here were obtained by a low level data fusion^25^ of the positive and negative ionisation modes by horizontal concatenation, and taking an average of the different lipid ions that were detected for a specific lipid class. For example, an average of the peak intensities of lysophosphatidylethanolamine LPE(16:0) − H, LPE(18:3) − H, LPE(18:2) − H, LPE(16:0) + H, LPE(18:3) + H and LPE(18:2) + H was taken to obtain the overall peak intensity of the LPE class.

Authentic standards, mostly plant based, from the AgResearch chemical inventory were run through the HILIC and C18 streams, under conditions identical to the current study. Parent masses in respective ionisation modes and retention times were listed in a table, and this library of authentic standards was matched against the corresponding final data matrices with tolerances of 5 ppm for parent mass and 3 s for retention time, to identify any hits. The libraries had 297, 170, 142 and 227 parent masses to match in C18 positive and negative, and HILIC positive- and negative-ionisation modes, and predominantly contained amino acids, secondary metabolites and their derivatives in C18, and amino acids, sugars, organic acids and their derivatives in HILIC streams, respectively.

*Statistical analysis*: Statistical analyses were performed using MetaboAnalyst ver 3.0, an online metabolomics analysis suite^69^. A data scaling procedure (auto-scaling) was carried out, where the data were normalised (mean-centred and divided by standard deviation of each variable) so that the features (peak intensities) are comparable.

Univariate and multivariate data analyses^70^ were conducted. For univariate analysis using *t* tests, features detected as significant with a false discovery rate cut-off of *p* < 0.05 were evaluated. For the multivariate approach, PCA was used to interrogate the data. Score plots which display the distribution of each sample along the composite variables of the score plot graph are presented.

Minitab 18 (Minitab Inc., USA) software was used to generate the interval plot (Fig. 1), cloud plot (Fig. 3), and boxplots (Fig. 2a, Fig.4); MS Excel (Microsoft Inc., USA) was used to generate Fig. 2c; and an online correlation analysis tool (www.sthda.com) was used to generate Fig. 2b.

*Pathway analysis and compound identification*: While eliminating redundant signals is vital for the identification of biomarkers, retaining these signals will facilitate network predictions^25^. Therefore, the raw data matrix obtained from the diffreport function (high- vs. low-sugar groups) of XCMS was used for pathway analysis. Network predictions/pathway analysis was performed using *Mummichog*^71^, a programme that combines metabolite prediction and network analysis in one step^72,73^. In contrast to the traditional approach, *Mummichog* first populates related spectral features to a network, with the hypothesis that if features reflect biological activity, then the metabolites they represent must exhibit enrichment in the local network. Metabolite identifications along with enriched pathway modules impart a broad understanding of tentative mechanisms, and provides scope for further probing. Input files for *Mummichog* from the HILIC and C18 streams comprised *m/z* values, retention time, *p* values and fold-change values. Due to its genetic synteny with ryegrass^74^, metabolic networks/pathways of barley (*Hordeum vulgare*) from the Plant Metabolic Network (PMN), www.plantcyc.org, December, 2016, were used as a reference. Appropriate parameters were selected for the ionisation mode, instrument (Orbitrap) and mandatory identification of parent masses, while other parameters were set as default.

Significant nodes of all four streams from respective activity networks were collated, and *m/z* features showing fold-change greater than 1.5 alone were selected. Features that matched authentic standards in the local library, and manually identified compounds that had fold-change greater than 1.5, were also added to this list. KEGG IDs for these shortlisted features were uploaded to KEGG Mapper^75^, http://www.kegg.jp/kegg/tool/map_pathway2.html, December, 2016. Modules directly involving the identified compounds with reference to rice (*Oryza sativa*) pathways, were displayed.

In addition to compounds identified with level 1 confidence^44^ by matching with a local library of authentic standards, *de novo* compound identification with level 2 confidence was performed as demonstrated by Subbaraj et al.^76^. Compounds identified by *Mummichog* were only given a level 3 status, a level that implies compound class.

*Pathway visualisation using MapMan*: MapMan uses a hierarchical ontology approach to visualise pathways and their corresponding modules in a functional context^77^. A customised map, based on the module/pathway data generated by KEGG Mapper, was built for the current study. The experimental data file comprised the compounds identified in Table 1, along with the respective *t* stat values. KEGG Mapper results were sorted into appropriate bins in accordance with MapMan syntax (Table 2) and added as the mapping file. Finally, a custom made picture (.bmp) that accommodates all the identified pathways and modules was added to the pathways folder. The ImageAnnotator module of MapMan uses mapping files as its data source, and then paints the input experimental data to the custom made images/maps according to the hierarchical structure of the mapping files^77^.

**Fig. 6:**
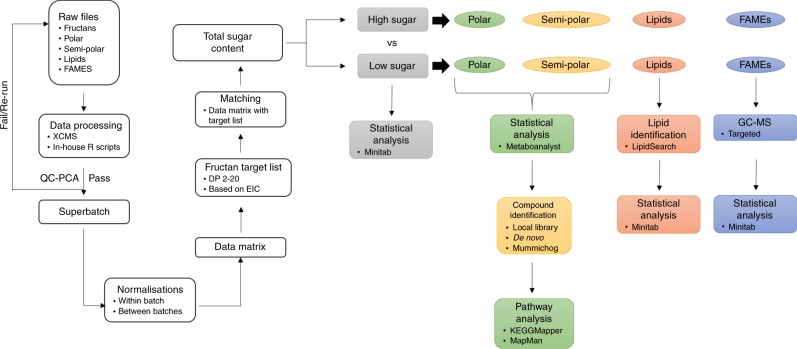
Summarised overview of data analysis from raw files of fructan data through to total sugar content, classification of high- and low-sugar groups, and subsequent analysis of fatty acid methyl esters (FAMEs), lipids, polar and semi-polar compounds

A summarised overview of data analysis is presented in Fig. 6.

## Supplementary information

Description of Supplementary Data

Supplementary Information

Supplementary data 1

Supplementary data 2

Supplementary data 3

## Data Availability

Raw data files (Thermo.raw files) are available at MetaboLights database^78^ (www.ebi.ac.uk/metabolights), under the following study IDs: Oligosaccharides (MTBLS 529), HILIC-positive ionisation mode (MTBLS 62), HILIC-negative ionisation mode (MTBLS 63), C18-positive ionisation mode (MTBLS 64), C18-negative ionisation mode (MTBLS 65), lipids-positive ionisation mode and FAMEs (MTBLS 66) and lipids-negative ionisation mode (MTBLS 68). Allied metadata was created using the ISA-creator package from MetaboLights, and comprised genotype, replicate, extraction, chromatography and instrument conditions, ionisation mode and mass range information.
