# Supplementary material for: A large-scale metabolomics study to harness chemical diversity and explore biochemical mechanisms in ryegrass
Source: Commun Biol. 2019 Mar 4;2:87. doi: 10.1038/s42003-019-0289-6 (PMC6399292; doi:10.1038/s42003-019-0289-6)
Supplement: Supplementary file 1 — Description of Supplementary Data [file 42003_2019_289_MOESM1_ESM.docx]

**Description of Additional Supplementary Files**

**File Name**: Supplementary Data 1

**Description**: Metabolic features of the HILIC (polar) and C18 (semi-polar) streams that were significantly different at a false-discovery rate cut-off of *p* < 0.05, between the high- (*n* = 133) and low-sugar (*n* = 106) groups, based on *t* tests. HP, HN, CP and CN denote different analytical streams corresponding to HILIC positive, negative and C18 positive and negative, respectively; A positive *t* stat value indicates high- > low-sugar group (↑), whereas a negative value indicates high- < low-sugar group (↓). HP_97.029_851.31 denotes a metabolic feature in HILIC positive ionisation mode with *m/z* 97.029 at retention time 851.31 secs.

**File Name**: Supplementary Data 2

**Description**: Lipid species in ryegrass identified by LipidSearch software in both positive and negative ionisation modes. *m/z* denotes mass-to-charge ratios of the features used for identification; RT denotes retention time in minutes; Fatty acid refers to tentative assignments of different fatty acids to the glycerol backbone. DG – diglyceride; DGDG – digalactosyldiacylglycerol; DGMG – digalactosylmonoacylglycerol; LPC – lysophosphatidylcholine; LPE – lysophosphatidylethanolamine; LPG – lysophosphatidylglycerol; MG – monoglyceride; MGDG – monogalactosyldiacylglycerol; MGMG – monogalactosylmonoacylglycerol; PA - phosphatidic acid; PC – phosphatidylcholine; PE – phosphatidylethanolamine; PG – phosphatidylglycerol; PI – phosphatidylinositol; Pme – phosphatidylmethanol; PS – phosphatidylserine; SQDG – sulfoquinovosyldiacylglycerol; TG – triglyceride.

**File Name**: Supplementary Data 3

**Description**: Average peak intensities and standard errors of low- and high-DP fructans in the 39 high-sugar genotypes. Data correspond to Figure 2c.
