# Supplementary material for: A large-scale metabolomics study to harness chemical diversity and explore biochemical mechanisms in ryegrass
Source: Commun Biol. 2019 Mar 4;2:87. doi: 10.1038/s42003-019-0289-6 (PMC6399292; doi:10.1038/s42003-019-0289-6)
Supplement: Supplementary file 2 — Supplementary Information [file 42003_2019_289_MOESM2_ESM.pdf]

Supplementary Information

Supplementary Figures

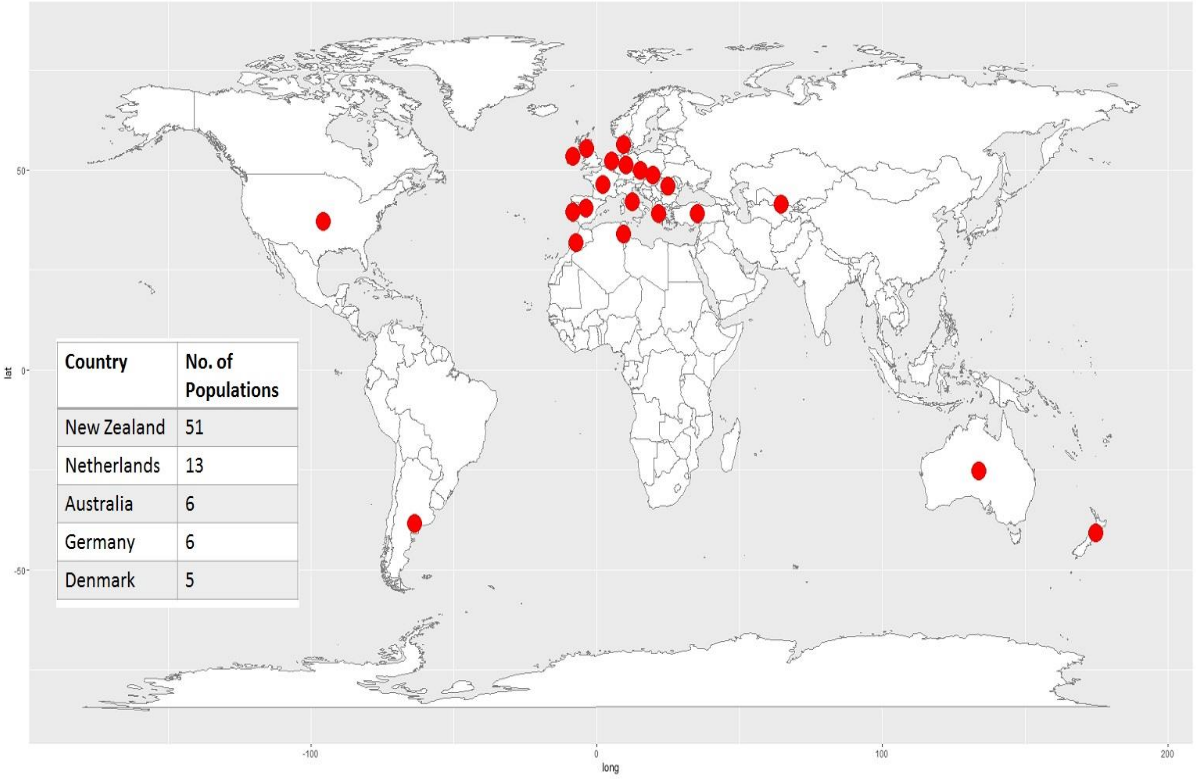

Supplementary Figure 1. Country-wise distribution of ryegrass populations used in the study, showing the top 5 countries that contributed maximum number of populations. Red dots refer to representative countries. Figure created using worldmap_plotting.R (https://gist.github.com/Ram-N/5296284#file-worldmap_plotting-r) with appropriate modifications.

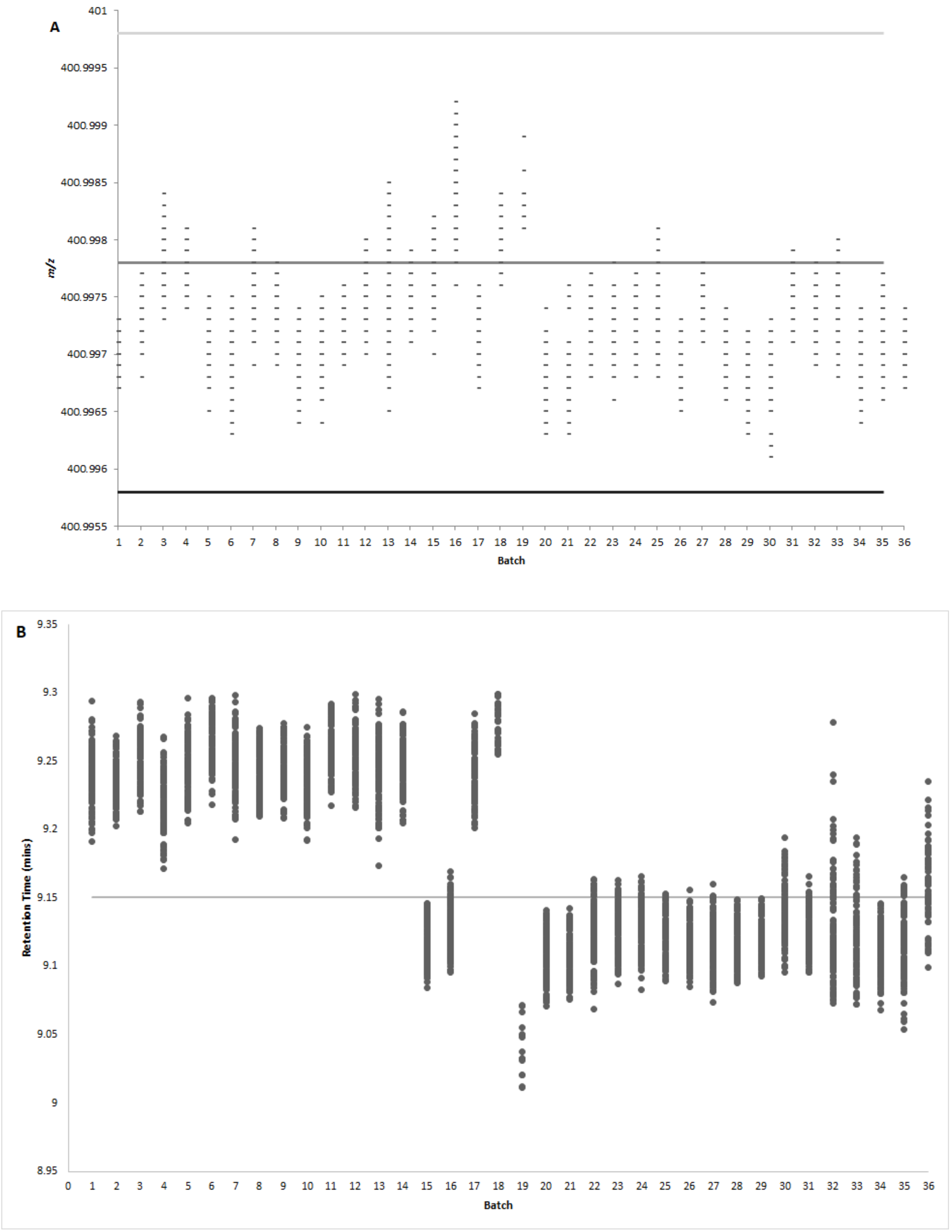

Supplementary Figure 2. Drifts in **(A)** mass accuracy (*m/z*) and **(B)** retention time (mins) of internal standard 2′,7′-Dichlorofluorescein in QC samples of C18 positive ionisation mode, across all 36 batches. In **(A)**, the middle grey line shows the exact mass of the internal standard (400.9978) with the upper (light grey) and lower (dark grey) limits of the ± 5 ppm range. In **(B)**, the middle grey line shows the median retention time (9.15 mins) of the internal standard.

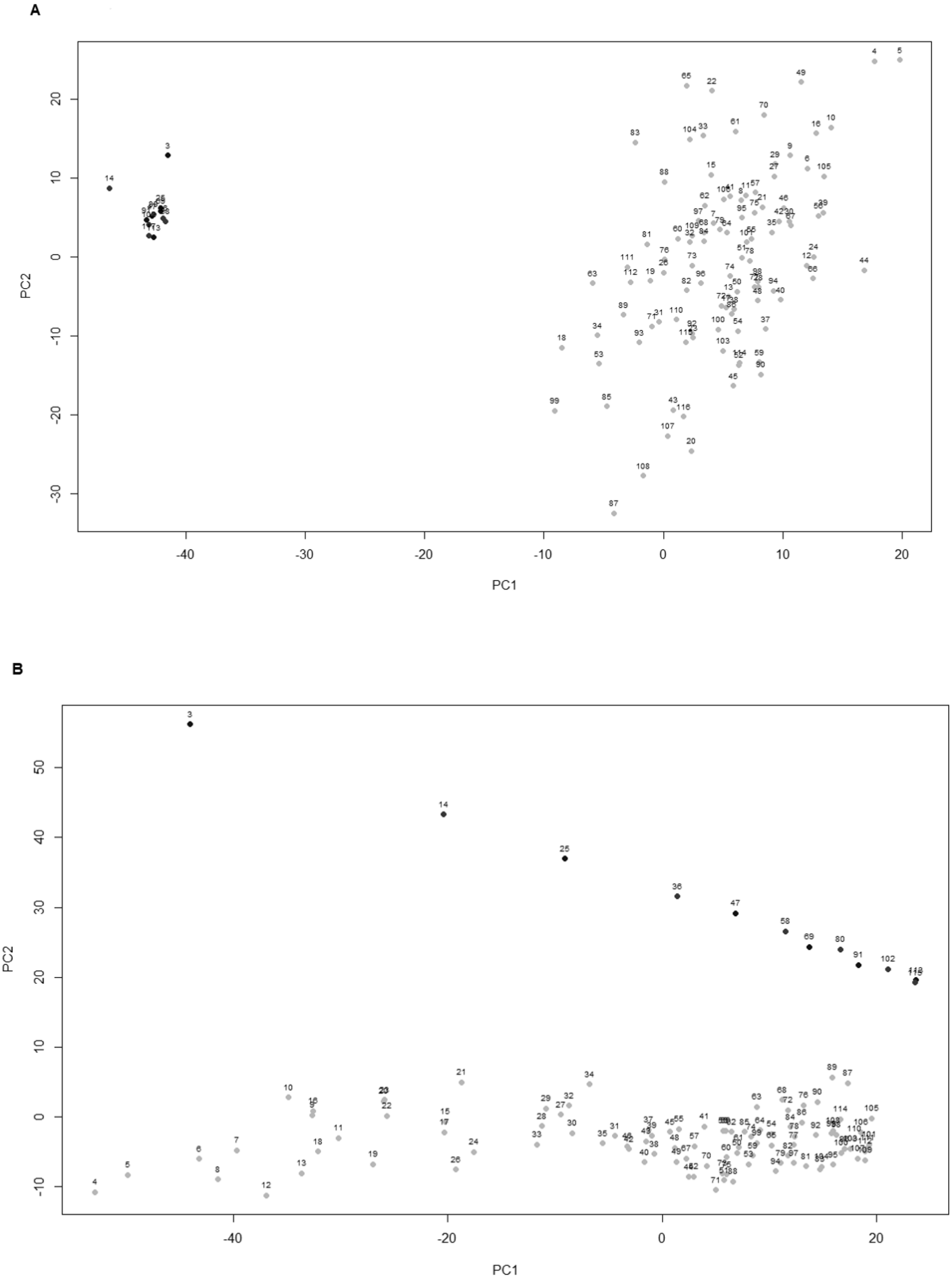

Supplementary Figure 3. Post-run QC evaluation of run-order effects within a batch from the C18 stream using principal component analysis (PCA), where **(A)** shows a batch with no apparent run-order effect, and **(B)** shows a batch with a notable run-order effect. Black dots with sample numbers represent QC samples, and grey dots represent samples in a batch.

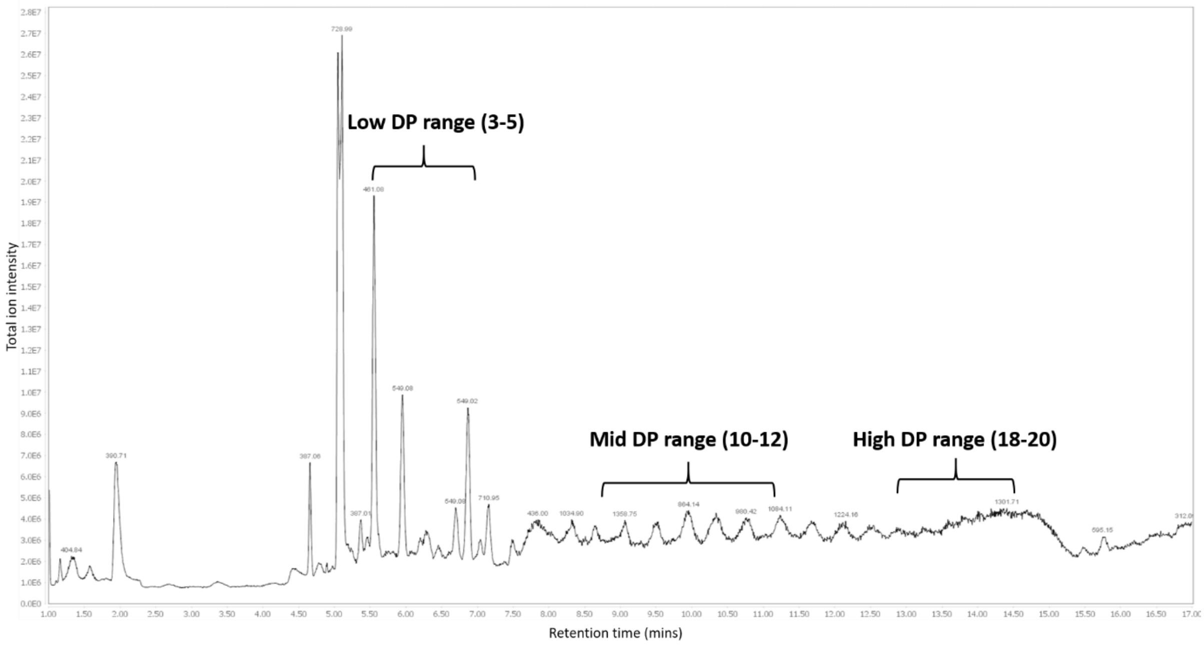

Supplementary Figure 4. A typical total ion chromatogram of a sample for fructan/sugar measurement, showing low (3-5), mid (10-12) and high (18-20) degree of polymerisation (DP) ranges.

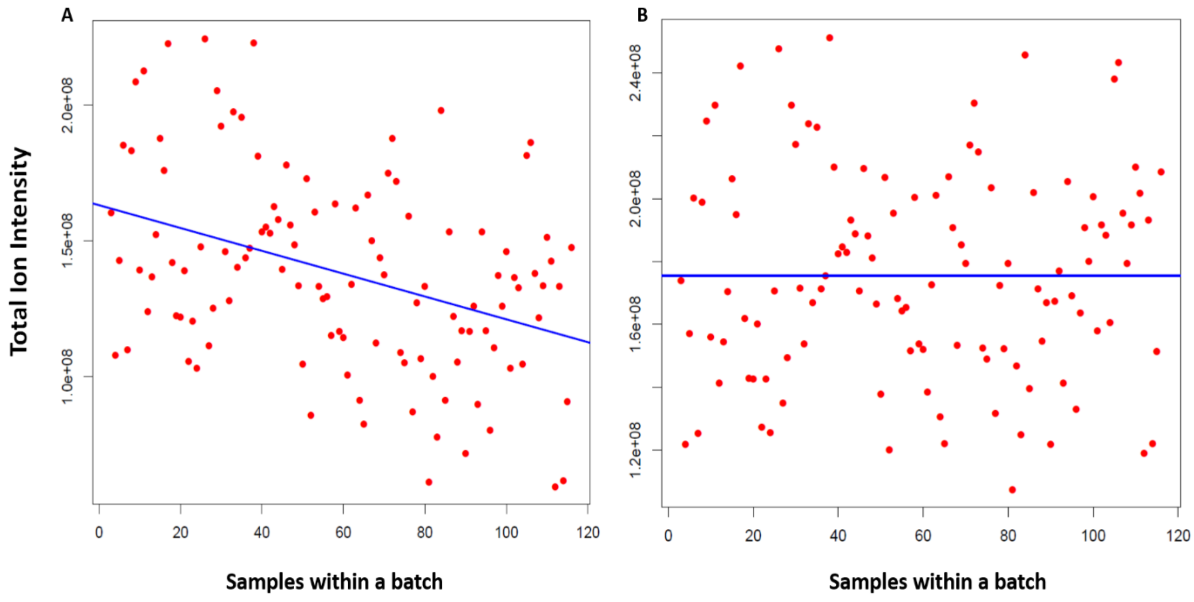

Supplementary Figure 5. Individual sample fructan/sugar estimates within a single batch, **(A)** before and **(B)** after normalisation by a linear trend. Red dots represent samples, and the blue line denotes the linear regression trend.

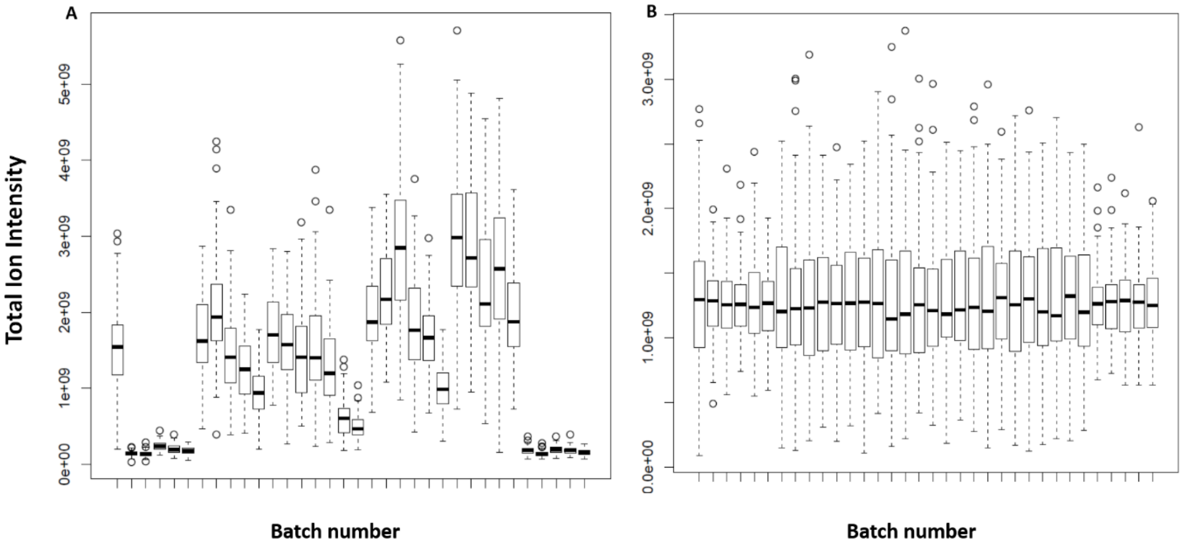

Supplementary Figure 6. Range of fructan/sugar estimates within all 36 batches **(A)** before and **(B)** after normalisation by comBat.

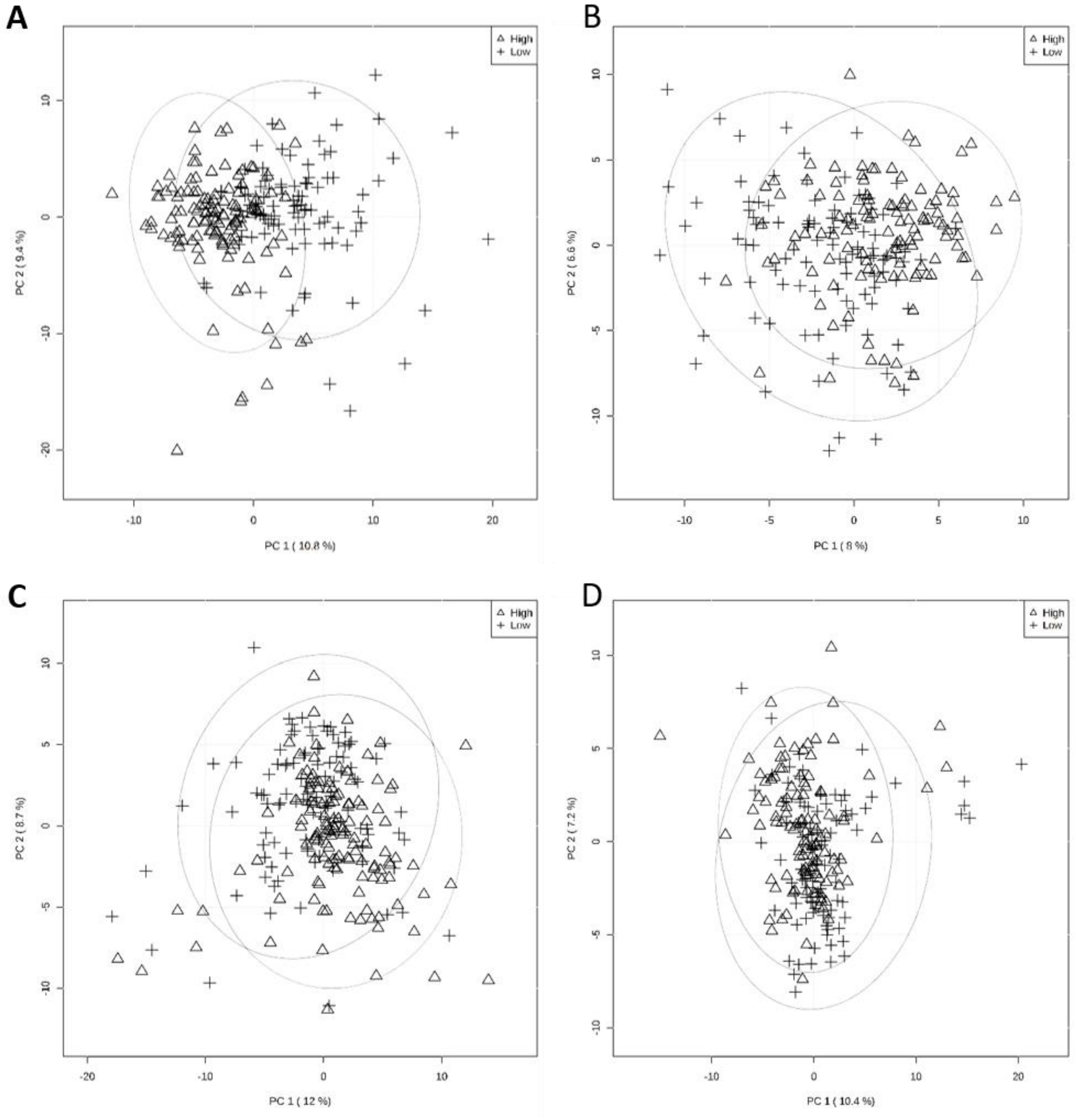

Supplementary Figure 7. Principal component analysis (PCA) of the four different analytical streams and their ionisation modes, **(A)** HILIC positive, **(B)** HILIC negative, **(C)** C18 positive and **(D)** C18 negative, between the high- (Triangle) and low-sugar (Plus) groups.

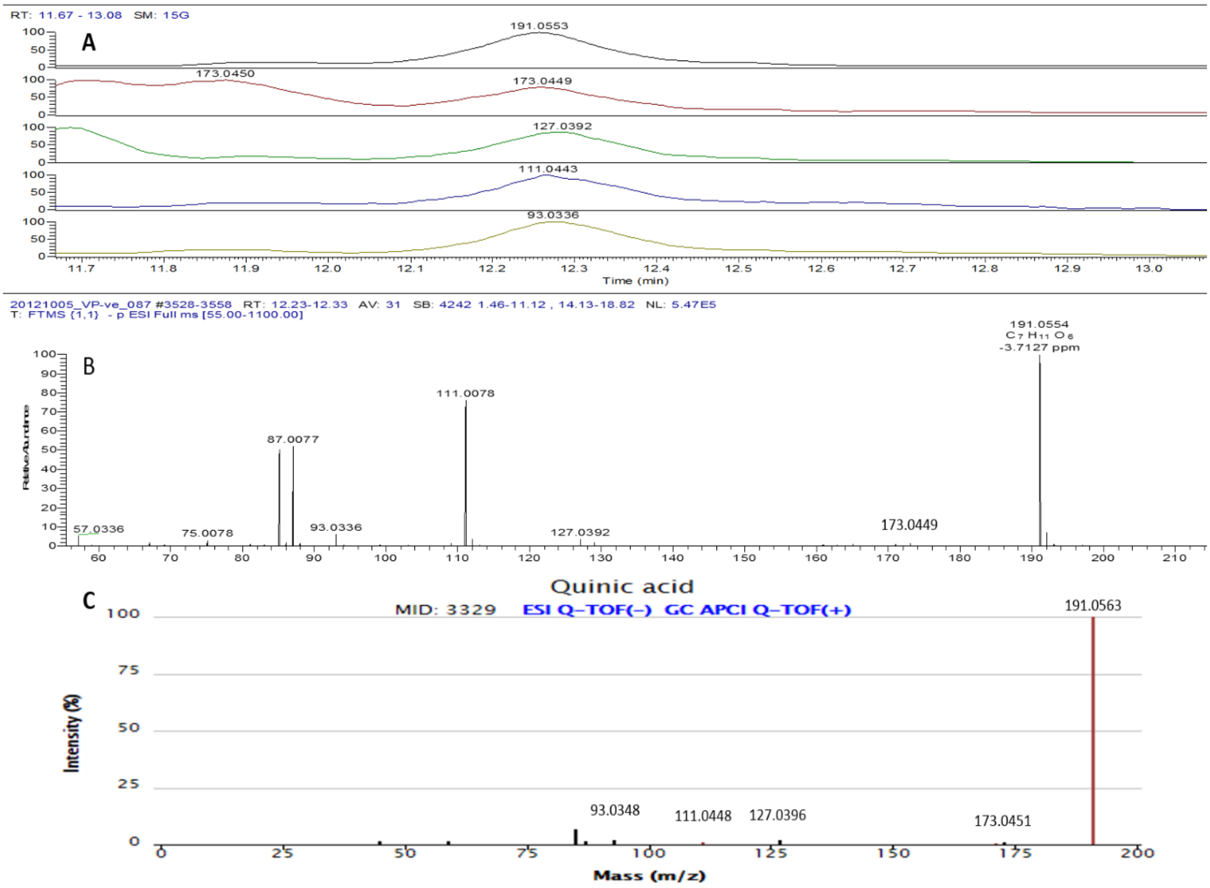

Supplementary Figure 8. Tentative identification of quinic acid in a sample run in HILIC negative ionisation mode, based on matching with spectral databases. **(A)** shows co-elution of extracted ion chromatograms corresponding to the parent mass *m/z* 191.0554 with those of diagnostic fragments *m/z* 173.0449, 127.0392, 111.0443 and 93.0336; **(B)** shows mass spectra of diagnostic fragments *m/z* 93.0336, 111.0078, 127.0392 and 173.0449 in the sample and mass error of the parent mass to the exact mass of quinic acid ($C_7H_{12}O_6$); and **(C)** shows diagnostic mass spectra of quinic acid in the METLIN database.

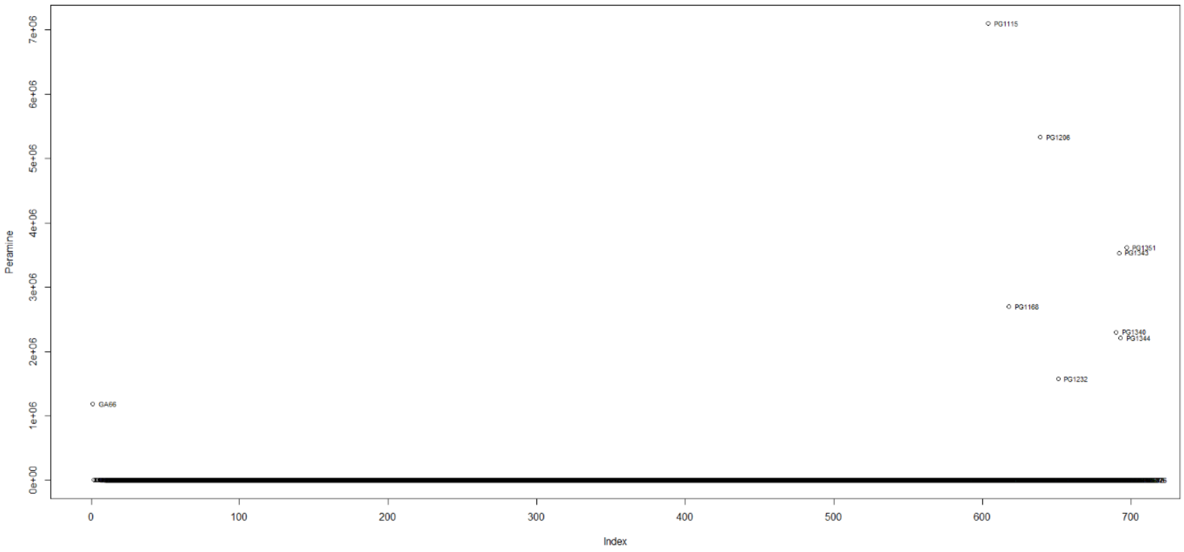

Supplementary Figure 9. Distribution of ryegrass genotypes based on detection of peramine (an endophyte-specific alkaloid). Nine genotypes that showed peramine presence were excluded from subsequent analyses.

Supplementary Tables

Supplementary Table 1. Selection of high- and low-sugar grasses based on total sugar content with corresponding genotypes, populations, brief description and representative countries.

Genotype	Population	Description	Country	Total sugar content
**PG0001**	GIL025	NZ elite breeding pool	NZ	High
**PG0020**	GIL014	NZ elite breeding pool	NZ	High
**PG0044**	GIL029	NZ elite breeding pool	NZ	High
**PG0074**	GIL028	NZ elite breeding pool	NZ	High
**PG0077**	GIL028	NZ elite breeding pool	NZ	High
**PG0098**	GIL028	NZ elite breeding pool	NZ	High
**PG0118**	GIL027	NZ elite breeding pool	Unknown	High
**PG0123**	GIL027	NZ elite breeding pool	Unknown	High
**PG0146**	Tolosa	NZ elite breeding pool	NZ	High
**PG0163**	GIL024	NZ elite breeding pool	NZ	High
**PG0218**	GIL022	NZ elite breeding pool	NZ	High
**PG0228**	GIL026	NZ elite breeding pool	NZ	High
**PG0285**	GIL009	NZ elite breeding pool	NZ	High
**PG0301**	GIL009	NZ elite breeding pool	NZ	High
**PG0372**	GIL005	NZ elite breeding pool	NZ	High
**PG0408**	GIL004	NZ elite breeding pool	NZ	High
**PG0445**	GIL002	NZ elite breeding pool	NZ	High
**PG0503**	*Aber*dart	UK high sugar cultivar	UK	High

**PG0520**	Aurora	UK high sugar cultivar	UK	High
**PG0548**	Bardonna	European cultivar	Netherlands	High
**PG0556**	Barlet	European cultivar	Netherlands	High
**PG0560**	Barlet	European cultivar	Netherlands	High
**PG0574**	Bronsyn	NZ cultivar	NZ	High
**PG0585**	Chantal	European cultivar	Denmark	High
**PG0598**	Corvair	US cultivar	Netherlands	High
**PG0675**	Grasslands Samson	NZ cultivar	NZ	High
**PG0688**	Hella	European cultivar	Denmark	High
**PG0697**	Hillary	NZ cultivar	NZ	High
**PG0701**	Hillary	NZ cultivar	NZ	High
**PG0743**	Liperry	European cultivar	Germany	High
**PG0747**	Liperry	European cultivar	Germany	High
**PG0753**	Liprior	European cultivar	Germany	High
**PG0808**	Medea	Australian cultivar	Australia	High
**PG0880**	Pastel	European cultivar	Slovakia	High
**PG0902**	Pippin	European cultivar	Unknown	High
**PG0917**	Premium	European cultivar	Netherlands	High
**PG0964**	Tolosa	NZ cultivar	NZ	High
**PG1074**	Banks	NZ cultivar	Unknown	High
**PG1293**	Tuni-9	Ecotype	Tunisia	High
**PG0005**	GIL014	NZ elite breeding pool	NZ	Low

**PG0013**	GIL014	NZ elite breeding pool	NZ	Low
**PG0038**	GIL012	NZ elite breeding pool	NZ	Low
**PG0122**	GIL027	NZ elite breeding pool	NZ	Low
**PG0208**	GIL022	NZ elite breeding pool	NZ	Low
**PG0413**	GIL004	NZ elite breeding pool	NZ	Low
**PG0417**	GIL004	NZ elite breeding pool	NZ	Low
**PG0628**	Epic	US cultivar	USA	Low
**PG0695**	Heraut	European cultivar	Netherlands	Low
**PG0698**	Hillary	NZ cultivar	NZ	Low
**PG0710**	Kerem	European cultivar	Germany	Low
**PG0717**	Limanda	Unknown	Unknown	Low
**PG0768**	Turk-12 x Commando AR1	NZ elite breeding pool	NZ	Low
**PG0778**	Manhattan	US cultivar	USA	Low
**PG0790**	Mara	European cultivar	Romania	Low
**PG0828**	Medea	Australian cultivar	Australia	Low
**PG0895**	Pearl	US cultivar	USA	Low
**PG0969**	Vedette	NZ cultivar	NZ	Low
**PG1010**	Portugal (Port-9)	Ecotype	Portugal	Low
**PG1036**	Fra-15	Ecotype	France	Low
**PG1041**	Australia (Aus-1)	Ecotype	Australia	Low

**PG1069**	Morroco (Mor-5)	Ecotype	Morocco	Low
**PG1109**	Greece<br>(Gre-1)	Ecotype	Greece	Low
**PG1173**	Vedette	NZ cultivar	NZ	Low
**PG1179**	Ruanui		Unknown	Low
**PG1210**	GIL020	NZ elite breeding pool	NZ	Low
**PG1280**	Tuni-2	Ecotype	Tunisia	Low
**PG1287**	Tuni-2	Ecotype	Tunisia	Low
**PG1288**	Tuni-2	Ecotype	Tunisia	Low
**PG1339**	Iran-3 x Commando AR1	NZ elite breeding pool	NZ	Low
**PG1361**	Turk-1 x Commando AR1	NZ elite breeding pool	NZ	Low

Total sugar content is the sum of hexose units of low (3, 4 and 5), mid (10, 11 and 12) and high (18, 19 and 20) degree of polymerisation (DP) ranges. Here, population is defined as a group of individuals of the same species with characteristics in common, including location, genetic relatedness, or intended use; cultivar (cultivated variety) is a population developed by selective breeding that is available as a product for on-farm use; an elite breeding pool is a group of individuals selected from a natural population for use in a breeding programme, which has undergone further selection for desirable traits; and an ecotype refers to a population that occurs naturally in a distinct, well-defined environment.

Supplementary Table 2. XCMS settings used for peak detection in the different analytical streams

**Fructans**
xset = xcmsSet(mzdatafiles, nSlaves = nSlve, method='matchedFilter', fwhm = 120, sigma = 120/2.3548, max = 5, snthresh = 10, step = 0.1, steps = 2, mzdiff = 0.8 - (0.1*2), index = FALSE, sleep = 0)
**Semi-polar compounds (UHPLC)**
xset = xcmsSet(mzXMLfiles, nSlaves = nSlve, method = "centWave", ppm = 10, mzdiff = 0.001, peakwidth =c(5,20), prefilter = c(3,10000), snthresh = 20, integrate = 1, noise = 5000)
**Lipids and polar compounds (HPLC)**
xset = xcmsSet(mzXMLfiles, nSlaves = nSlve, method = "centWave", ppm = 10, mzdiff = 0.001, peakwidth =c(10,60), prefilter = c(3,10000), snthresh = 20, integrate = 1, noise = 5000)

Supplementary Table 3. An exemplar of fructan peak annotations and measurements for DP3

**Parameters for DP3 in the target list**						**DP3 measurements in sample**		
**ID**	**DP**	***m/z***	**rt**	**rtmin**	**rtmax**	**Peak intensity**	**DP3 Sum**	**Sum of hexose units**
DP03_Mz	3	503.1618	270	270	480	6597106	32523436	32523436 × 3 = 97570308
DP03_MFA	3	549.1673	270	270	480	20235466		
DP03_MCl	3	539.1379	270	270	480	4864410		
DP03_Dimer	3	1007.3309	270	270	480	826453.9		

Mz, MFA, MCl and Dimer represent the parent mass, formic acid and chlorine adducts, and the dimer respectively. rt, rtmin and rtmax represent the expected retention time of DP3 with corresponding lower and upper limits in seconds, respectively.

Supplementary Table 4. List of lipids identified by LipidSearch software

Group	Lipid name
**P-Choline**	Lysophosphatidylcholine
	Platelet-activating factor
	Phosphatidylcholine
**P-Ethanol Amine**	Lysophosphatidylethanolamine
	Lysodimethylphosphatidylethanolamine
	Phosphatidylethanolamine
	Dimethylphosphatidylethanolamine
**P-Serine**	Lysophosphatidylserine
	Phosphatidylserine
**P-Glycerol**	Lysophosphatidylglycerol
	Phosphatidylglycerol
**P-Inositol**	Lysophosphatidylinositol
	Phosphatidylinositol
**P-Ethanol**	Lysophosphatidylethanol
	Phosphatidylethanol
**P-Acid**	Lysophosphatidic acid
	Phosphatidic acid
	Cyclic phosphatidic acid
**P-Methanol**	Lysophosphatidylmethanol
	Phosphatidylmethanol
**Sphingolipids**	Sphingomyelin
	Lysosphingomyelin
	Sphingomyelin(phytosphingosine)
**Neutral glycerolipid**	Monoglyceride
	Diglyceride
	Triglyceride
**Fatty Acid**	Fatty acid
	(O-acyl)-1-hydroxy fatty acid
**Cardiolipin**	Cardiolipin
**Sphingoid base**	Sphingoshine
	Sphingoshine phosphate
**Neutral Glycosphingolipids**	Glucosylsphingoshine
	Simple glc series

**Glycosphingolipids**	Ceramides
	Ceramides phosphate
	Gangliosides
**Steroid**	Cholesteryl ester
	Zymosteryl
	Stigmasteryl ester
	Sitosteryl ester
	Deuterated cholesteryl ester
**Coenzyme**	Coenzyme
**Glycoglycerolipid**	Monogalactosylmonoacylglycerol
	Monogalactosyldiacylglycerol
	Digalactosylmonoacylglycerol
	Digalactosyldiacylglycerol
	Sulfoquinovosylmonoacylglycerol
	Sulfoquinovosyldiacylglycerol
**Neutral glycerolipid (deuterated)**	Deuterated diglyceride
	Deuterated triglyceride

Supplementary Methods

Trial conditions

Seeds of 724 genotypes were germinated in cell trays with a peat based potting mix under ventilated glasshouse conditions. At the five tiller stage, seedlings were transplanted to planter bags. After 60 days, the plant was divided into five clonal replicates and planted in five separate planter bags (clonal replicates). Clonal replicates were grown in planter bags with a peat and pumice based commercial growing medium (Daltons, New Zealand). The plants were evenly spaced (approximately 35 cm) on a concrete pad, under natural conditions at AgResearch Limited, Palmerston North, New Zealand (40°22'50.3"S, 175°36'53.2"E). Irrigation was provided every night. The fertiliser regime comprised application of approximately 2g of Ravensdown Nitrophoska Extra (Ravensdown, New Zealand) and Everris Osmocote (Everris International B.V., The Netherlands) per bag, once in 40 and 90 days, respectively. Other agronomic practices such as weeding were done as and when required.
